# Mapping the Response of Human Osteocytes in Native Matrix to Mechanical Loading Using RNA Sequencing

**DOI:** 10.1002/jbm4.10721

**Published:** 2023-02-21

**Authors:** Chen Zhang, Huib W. van Essen, Daoud Sie, Dimitra Micha, Gerard Pals, Jenneke Klein‐Nulend, Nathalie Bravenboer

**Affiliations:** ^1^ Academic Centre for Dentistry Amsterdam (ACTA), University of Amsterdam and Vrije Universiteit Amsterdam, Department of Oral Cell Biology Amsterdam Movement Sciences Amsterdam The Netherlands; ^2^ Amsterdam University Medical Centers (AUMC)/Location VUmc, Vrije Universiteit Amsterdam, Department of Clinical Chemistry Amsterdam Movement Sciences Amsterdam The Netherlands; ^3^ Amsterdam University Medical Centers (AUMC)/Location VUmc, Vrije Universiteit Amsterdam, Department of Human Genetics Amsterdam Movement Sciences Amsterdam The Netherlands

**Keywords:** 3D, HUMAN BONE, MECHANICAL LOADING, NATIVE MATRIX, OSTEOCYTES, RNA SEQUENCING

## Abstract

Osteocytes sense mechanical loads and transduce mechanical signals into a chemical response. They are the most abundant bone cells deeply embedded in mineralized bone matrix, which affects their regulatory activity in the mechanical adaptation of bone. The specific location in the calcified bone matrix hinders studies on osteocytes in the in vivo setting. Recently, we developed a three‐dimensional mechanical loading model of human osteocytes in their native matrix, allowing to study osteocyte mechanoresponsive target gene expression in vitro. Here we aimed to identify differentially expressed genes by mapping the response of human primary osteocytes in their native matrix to mechanical loading using RNA sequencing. Human fibular bone was retrieved from 10 donors (age: 32–82 years, 5 female, 5 male). Cortical bone explants (8.0 × 3.0 × 1.5 mm; length × width × height) were either not loaded or mechanically loaded by 2000 or 8000 μɛ for 5 minutes, followed by 0, 6, or 24 hours post‐culture without loading. High‐quality RNA was isolated, and differential gene expression analysis performed by R2 platform. Real‐time PCR was used to confirm differentially expressed genes. Twenty‐eight genes were differentially expressed between unloaded and loaded (2000 or 8000 μɛ) bone at 6 hours post‐culture, and 19 genes at 24 hours post‐culture. Eleven of these genes were related to bone metabolism, ie, *EGR1*, *FAF1*, *H3F3B*, *PAN2*, *RNF213*, *SAMD4A*, and *TBC1D24* at 6 hours post‐culture, and *EGFEM1P*, *HOXD4*, *SNORD91B*, and *SNX9* at 24 hours post‐culture. Mechanical loading significantly decreased *RNF213* gene expression, which was confirmed by real‐time PCR. In conclusion, mechanically loaded osteocytes differentially expressed 47 genes, of which 11 genes were related to bone metabolism. RNF213 might play a role in mechanical adaptation of bone by regulating angiogenesis, which is a prerequisite for successful bone formation. The functional aspects of the differentially expressed genes in bone mechanical adaptation requires future investigation. © 2023 The Authors. *JBMR Plus* published by Wiley Periodicals LLC on behalf of American Society for Bone and Mineral Research.

## Graphical abstract

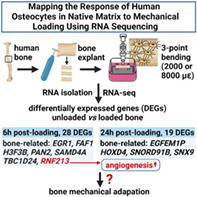

## Introduction

Osteocytes are the most abundant cells in bone, comprising 90% to 95% of all bone cells.^(^ ^1^ ^)^ Their cell bodies reside in lacunae and their processes are running through canaliculi forming an extensive lacuno‐canalicular network throughout the bone, thereby connecting osteocytes to each other, osteoblasts, bone surface lining cells, vasculature, and bone marrow.^(^ ^2^, ^3^, ^4^ ^)^ The specific location of osteocytes deeply embedded in bone, combined with their extensive distribution throughout bone, enables osteocytes to function as the professional mechanosensory cells of bone.^(^ ^4^ ^)^ Osteocytes transduce mechanical signals into a biochemical response,^(^ ^5^ ^)^ resulting in the regulation of osteoblastic bone formation and/or osteoclastic bone resorption.^(^ ^6^, ^7^ ^)^ It is generally accepted that mechanical loading deforms the bone matrix, thereby generating a pressure gradient in the interstitial fluid surrounding osteocytes.^(^ ^8^, ^9^ ^)^ The osteocytes sense the pressure gradient‐induced fluid shear stress on their cell processes, and start to produce signaling molecules to regulate the activity of osteoblasts and/or osteoclasts.^(^ ^10^, ^11^ ^)^

The pericellular matrix surrounding osteocytes consists of proteoglycans, hyaluronic acid, and transverse elements.^(^ ^3^ ^)^ The proteoglycan perlecan in the pericellular matrix plays a role in maintaining the size of the pericellular space in the lacuno‐canalicular network. The fibers in the pericellular matrix act as sensing antennae of osteocytes.^(^ ^12^, ^13^ ^)^ The osteocyte processes are tethered to the canalicular wall by transverse fibrils, which are tension elements positioning the cell processes at the center of the canaliculus.^(^ ^3^ ^)^ The mechanical stimuli that can induce a cellular response require a certain magnitude of strain, which would cause bone fracture if it is directly induced by matrix deformation.^(^ ^14^ ^)^ Oscillatory fluid flow and pulsating fluid flow, but not substrate deformation of the same magnitude, induces a cellular response in osteocytes, indicating that fluid flow‐derived mechanical stimuli as a result of deformation are sensed by osteocytes, but not bone tissue deformation directly.^(^ ^15^, ^16^ ^)^ When bone is loaded, the flow of interstitial pericellular fluid induces a drag force on the matrix fibers, which is transmitted to the osteocyte's intracellular actin cytoskeleton.^(^ ^14^ ^)^ In this way, the mechanically induced strains can be greatly amplified at the osteocyte cell membrane level.^(^ ^14^ ^)^

Initial integrin‐mediated signaling events occur on a millisecond scale.^(^ ^17^, ^18^ ^)^ Moreover, mechanosensitive ion channels, G protein‐coupled mechanoreceptors, and focal adhesion signaling molecules, such as focal adhesion kinase, may play a role in the mechanosensation by osteocytes.^(^ ^19^ ^)^ The first signaling events are increased intracellular Ca^2+^ and release of adenosine triphosphate (ATP), which occur within 1 minute after initiation of mechanical stimulation.^(^ ^19^ ^)^ Then osteocytes release nitric oxide (NO)^(^ ^20^ ^)^ and prostaglandins (PGs),^(^ ^16^ ^)^ which regulate osteoblast and/or osteoclast activity.^(^ ^10^, ^11^ ^)^ Cyclooxygenase (COX) is the key enzyme in prostaglandin synthesis.^(^ ^21^ ^)^ COX‐2 has been shown to mediate the anabolic response of bone to mechanical loading.^(^ ^22^ ^)^ Canonical Wnt signaling plays an important role in regulating bone formation in response to mechanical loading in mice.^(^ ^23^ ^)^ The Wnt co‐receptor lipoprotein receptor‐related protein 5 (Lrp5) is crucial for osteoblast function in skeletal mechanotransduction.^(^ ^24^ ^)^ Sclerostin, an antagonist of Wnt signaling, binds to Lrp5 and inhibits bone formation by osteoblasts.^(^ ^25^ ^)^ Sclerostin, which is encoded by the *SOST* gene, is produced specifically by osteocytes.^(^ ^26^ ^)^ *SOST* gene expression and sclerostin production are decreased by mechanical loading in osteocytes.^(^ ^27^ ^)^ Osteocyte apoptosis is inhibited by pulsating fluid flow,^(^ ^28^ ^)^ whereas disuse or lack of mechanical loading promotes osteocyte apoptosis.^(^ ^29^ ^)^ Apoptotic osteocytes cause osteoclastogenesis to initiate bone remodeling to repair bone microdamage.^(^ ^30^ ^)^

Osteocytes are capable of modifying their own surrounding matrix.^(^ ^31^ ^)^ This matrix‐modifying activity is regulated by mechanical stimuli.^(^ ^32^ ^)^ Mechanical loading significantly affects gene expression of dentin matrix protein 1 (*DMP1*),^(^ ^31^ ^)^ and matrix extracellular phosphoglycoprotein (*MEPE*) in osteocytes.^(^ ^33^ ^)^ *DMP1* plays a distinct role in the attachment of osteocytes to the canalicular and lacunar wall.^(^ ^34^ ^)^ DMP1 regulates the mechanical signals sensed by osteocytes by changing the perilacunar matrix.^(^ ^31^ ^)^ Mechanical loading upregulates *MEPE* expression in osteocytes to inhibit osteoclastogenesis.^(^ ^33^ ^)^ Moreover, MEPE‐derived acidic, serine‐ and aspartic acid–rich motif (ASARM) inhibits mineralization of the peri‐lacunar matrix.^(^ ^35^ ^)^ MEPE, DMP1, and phosphate‐regulating gene with homologies to endopeptidases on X chromosome (PHEX) locally affect fibroblast growth factor 23 (FGF23) production.^(^ ^36^ ^)^ FGF23 is specifically expressed by osteocytes to regulate mineralization and phosphate homeostasis.^(^ ^37^ ^)^ *FGF23* messenger RNA (mRNA) is decreased by mechanical loading in osteocytes.^(^ ^38^ ^)^ Taken together, osteocytes change their microenvironment in response to mechanical loading, which in turn affects their mechanosensation and mechanotransduction, and ultimately the mechanical adaptation of bone.

Osteocyte cell lines and primary cells are used to study osteocytes in vitro because of their high availability and ease of use.^(^ ^7^ ^)^ Cell lines have their limitations, because they are modified to enable proliferation, whereas osteocytes in vivo do not proliferate.^(^ ^39^ ^)^ Osteocyte cell lines are mostly cultured in two‐dimensional (2D)‐monolayer, which lacks a pericellular matrix as is present around osteocytes in vivo. Cells cultured in 2D‐monolayer are flattened,^(^ ^5^ ^)^ whereas three‐dimensional (3D)‐osteocytes in bone are round and housed in ellipsoidal lacunae.^(^ ^40^ ^)^ Round nonadherent osteocytes are more mechanosensitive than flat adherent osteocytes.^(^ ^41^ ^)^ Round cellular morphology in their natural 3D‐conformation supports a less stiff cytoskeleton compared to the flat cells in monolayer culture, which benefits osteocytes in 3D to sense small strains.^(^ ^41^ ^)^ Osteocytes cultured in 2D also show less cell processes than when cultured in 3D.^(^ ^42^ ^)^ Osteocytes sense the mechanical loading by their cell processes.^(^ ^9^ ^)^ Osteocytes cultured in 3D demonstrate a superior phenotype compared to 2D‐cultured osteocytes.^(^ ^43^ ^)^ Human primary osteocytes cultured in 2D express low *SOST* and do not express *FGF23*, whereas 3D cultured cells on biphasic calcium phosphate microbeads increases expression of *SOST* and *FGF23*.^(^ ^44^ ^)^ The known inhibition of *SOST* and stimulation of *RANKL/OPG* gene expression by parathyroid hormone (PTH) is only observed in 3D, but not in 2D‐cultured human primary osteocytes.^(^ ^44^ ^)^ Besides the 3D‐cell network, oxygen tension may also influence the osteocyte phenotype.^(^ ^45^ ^)^ The oxygen concentration in the blood vessels in cortical bone is 4.2% (pO_2_ = 31.8 mmHg).^(^ ^46^ ^)^ Low oxygen tension (5%; hypoxia) promotes osteoblast differentiation toward osteocytes.^(^ ^47^ ^)^ Hypoxia facilitates the maintenance of an osteocyte phenotype in 2D‐monolayer culture with a distinct morphology than in normoxia.^(^ ^45^ ^)^ Hypoxia enhances sclerostin expression, but decreases alkaline phosphatase (ALP) activity compared with normoxia in primary human osteocytes.^(^ ^45^ ^)^

Recently, we established a 3D‐mechanical loading model to study osteocyte behavior in their native matrix in vitro.^(^ ^48^ ^)^ Mechanical loading at different magnitudes can be reliably applied on vital bone explants using three‐point bending.^(^ ^48^ ^)^ The osteocytes in the loaded bone explants keep their specific phenotype, and are able to respond to mechanical loading.^(^ ^48^ ^)^ This 3D‐mechanical loading model of bone explants in vitro mimics osteocytes in their native microenvironment in vivo.^(^ ^48^ ^)^ To date, the mechanism of osteocyte‐orchestrated adaptation of bone to mechanical loading is still not fully understood. Therefore, more insight into target gene expression by osteocytes in their native matrix in response to mechanical loading is necessary. Here we aimed to identify mechanosensitive genes by mapping the response of osteocytes in their native matrix to mechanical loading using RNA sequencing (RNA‐seq). The identified mechanosensitive genes might provide new insight into the mechanism of osteocyte mechanosensation and transduction. By further testing the function of the protein products of these genes, a better understanding of mechanical adaptation of bone might be achieved.

## Materials and Methods

### Human bone retrieval and mechanical loading

**Fig. 1:**
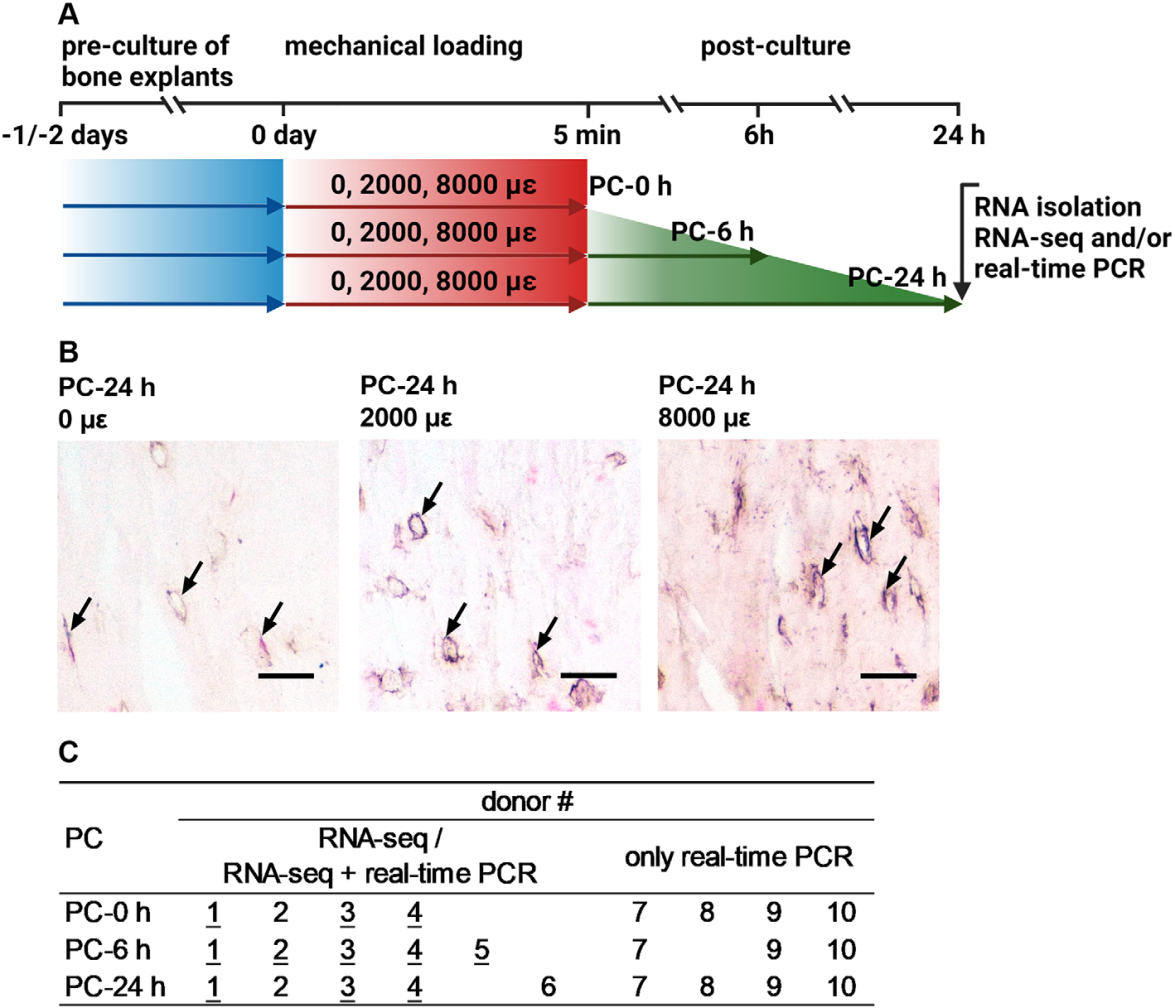
Schematic diagram of the experimental setup. (*A*) Bone explants were pre‐cultured for 1 or 2 days, and then were either not loaded or mechanically loaded at a magnitude of 2000 or 8000 μɛ for 5 minutes. Immediately after mechanical loading, explants were post‐cultured (without mechanical loading) for 0, 6, or 24 hours. RNA was isolated from osteocytes in bone and gene expression was measures by RNA‐seq and/or real‐time PCR. (*B*) Osteocytes expressing sclerostin in unloaded bone, and bone loaded at 2000 and 8000 μɛ at 24 hours post‐culture. Arrows: sclerostin‐positive osteocytes. Scale bar = 20 μm. (*C*) Donor number and indication of RNA‐seq and/or real‐time PCR at 0, 6, and 24 hours post‐culture. Not underlined: RNA‐seq or real‐time PCR only; Underlined: RNA‐seq + real‐time PCR. PC = post‐culture; RNA‐seq = RNA‐sequencing.

Fibular bone was collected from 10 white donors (age: 32–82 years, 5 female, 5 male; Table 1). The donors presented no medical history of skeletal pathology or trauma. All bone tissue was collected as surgical waste during mandible reconstruction surgery and obtained with donors' consent, and all protocols were approved by the local Medical Ethical Committee of the Amsterdam University Medical Centers (2016.105). Cortical fibular bone explants were washed in Hanks' balanced salt solution (HBSS; Thermo Fisher Scientific, Waltham, MA, USA). They were incubated in minimal essential medium (MEM; Thermo Fisher Scientific) containing 2 mg/mL type II collagenase (Worthington Biochemical, Lakewood, CA, USA) for 2 hours in a shaking water bath at 37°C, and washed again twice with HBSS. Then soft tissue was removed by scraping. Cleaned cortical bone explants were cut into small explants measuring 8.0 × 3.0 × 1.5 mm (length × width × height) using a diamond disc H‐345‐220 (Horico, Berlin, Germany), a handpiece (KaVo, Biberach an der Riss, Germany), and a foot control (KaVo), as described.^(^ ^48^ ^)^ Explants were cooled during cutting in ice‐cold HBSS. They were pre‐cultured for 1 or 2 days in six‐well plates (Merck KGaA, Darmstadt, Germany), containing MEM supplemented with 5% fetal bovine serum (FBS; Lonza BioWhittaker, Basel, Switzerland), 5% bovine calf serum (BCS; Thermo Fisher Scientific), 1% penicillin–streptomycin (10,000 U/mL; Thermo Fisher Scientific), and 0.5% amphotericin B solution (Merck KGaA, Darmstadt, Germany) at 37°C (Fig. 1*A*). Osteocyte viability and sclerostin expression are similar immediately after isolation and culture up to 2 days.^(^ ^48^ ^)^ Thus, osteocyte mechanoresponsiveness is unlikely to be changed by simply putting the bone pieces in culture. Explants were either not loaded or mechanically loaded by three‐point bending with a rounded contact point and a span length of 8 mm, at a magnitude of 2000 or 8000 μɛ at 1 Hz frequency for 5 minutes, as described.^(^ ^48^ ^)^ In short, mechanical loading was applied using a custom‐made loading apparatus, which generated sinusoidal displacement using a computer‐driven voice coil linear microactuator (type NCM04‐25250‐2LVE; H2W Technologies, Valencia, CA, USA).^(^ ^48^ ^)^ Mechanical loading at 2000 μɛ mimics physiological loading,^(^ ^49^, ^50^ ^)^ and 8000 μɛ mimics pathological overloading.^(^ ^51^ ^)^ The osteocytes in bone explants exposed to mechanical loading at 1600 μɛ are highly viable at 24 hours post‐culture, as shown in our previous study.^(^ ^48^ ^)^ Therefore, osteocytes in bone explants exposed to mechanical loading at 2000 μɛ, which is in the same physiological range, are expected to be highly viable as well. Mechanical loading at 8000 μɛ has been shown to increase osteocyte apoptosis in 8000 μɛ‐loaded rat ulnae.^(^ ^52^ ^)^ Therefore, we assumed that osteocyte viability was decreased in 8000 μɛ‐loaded human cortical bone in our study. Sclerostin expression and gene expression of *FGF23* and *SOST* are detected in osteocytes in bone explants, showing that they maintain their osteocytic phenotype.^(^ ^48^ ^)^ Immediately after mechanical loading, explants were post‐cultured (without mechanical loading) for 0, 6, or 24 hours in MEM containing 5% FBS, 5% BCS, 1% penicillin–streptomycin, and 0.5% amphotericin B solution (Fig. 1*A*). Explants collected at 24 hours post‐culture (not loaded or mechanically loaded by 2000 or 8000 μɛ for 5 minutes at 1 Hz) were embedded in MMA, cut into 5‐μm‐thick sections, and used for sclerostin immunostaining (Fig. 1*B*), as described.^(^ ^48^ ^)^

**Table 1:** Demographic Data of Bone Donors

Donor #	Gender ♂/♀	Age (years)	Disease
1	♂	61	Squamous cell carcinoma
2	♀	79	Squamous cell carcinoma
3	♂	74	Squamous cell carcinoma
4	♀	82	Carcinoma (margins of tongue)
5	♀	71	Squamous cell carcinoma
6	♀	69	Carcinoma (floor of mouth)
7	♂	61	Carcinoma (floor of mouth)
8	♂	79	Carcinoma (oral cavity)
9	♀	57	Squamous cell carcinoma
10	♂	32	Spindle cell rhabdomyosarcoma

Donor # = donor number; Gender ♂/♀ = male/female.

### Pulverization of bone explants

After mechanical loading, explants were instant frozen in liquid nitrogen. Three bone explants from the same donor were mechanically loaded at the same magnitude, and post‐cultured (without mechanical loading) for the same time period. Then explants were frozen in liquid nitrogen and stored at −80°C in 1.5 mL precooled (−80°C) RNAlater‐ICE (Thermo Fisher Scientific) for 2–9 weeks. Upon thawing, the three bone explants were pooled, weighed, put into one polycarbonate grind vial (SPEX SamplePrep, Metuchen, NJ, USA), and kept in liquid nitrogen. Twenty‐five milliliters TRIzol (Thermo Fisher Scientific)/g bone was frozen to pellets by dripping into liquid nitrogen, and added to bone explants. Bone explants were pulverized using a 6775 Freezer/Mill cryogenic grinder (SPEX SamplePrep, Metuchen, NJ, USA) filled with liquid nitrogen. Explants were grinded (rate: 10 cycles/s; 20 impacts/s) for 2 minutes in liquid nitrogen. After pulverization, the TRIzol/bone powder mixture was incubated for 1 hours in a shaking water bath at 37°C. Thereafter, RNA was isolated immediately, or the TRIzol/bone powder mixture was stored at −80°C before RNA isolation as described in RNA Isolation.

### RNA isolation

Two hundred microliters (200 mL) chloroform was added per microliter TRIzol/bone powder mixture, followed by 15 minutes centrifugation at 12,000*g* at 4°C. Isolation and purification of RNA was performed using RNeasy Midi columns (Qiagen, Hilden, Germany) or Zymo‐Spin IIICG columns (Direct‐zol RNA MiniPrep Plus; Zymo Research, Irvine, CA, USA). The supernatants were collected, mixed with an equal volume of 70% ethanol, and transferred to RNeasy Midi columns, or the supernatants were mixed with an equal volume of 100% ethanol, and transferred to Zymo‐Spin IIICG columns. RNA isolation and DNase I digestion were performed following the manufacturer's instructions. The RNA concentration was determined by Nanodrop spectrophotometer (Nanodrop Technologies, Wilmington, DE, USA) and Qubit RNA HS assay kits (Thermo Fisher Scientific). RNA quality was tested using the Agilent RNA 6000 Pico Assay performed in the Agilent 2100 Bioanalyzer system (Agilent Technologies, Santa Clara, CA, USA), presented as RNA integrity number (RIN). RNA samples with a RIN ≥ 7 or with two clear, distinct peaks of 18 S and 28 S ribosomal RNA on the electropherogram, and a yield ≥400 ng were acceptable for RNA sequencing.

### Complementary DNA library preparation, RNA‐seq, and data processing

Complementary DNA (cDNA) libraries were prepared from RNA samples from six donors (Fig. 1*C*) using KAPA mRNA Hyperprep kit (Roche Sequencing Solutions, Indianapolis, IN, USA), followed by sequencing on the Illumina HiSeq 4000 platform (Illumina, San Diego, CA, USA) to generate approximately 40 million 50‐base reads per sample. Sequence reads were quality trimmed using Sickle (version 1.33), and aligned to the GRCh37 reference (grch37_snp_tran) using HiSat2 (version 2.0.4). Resulting bam files were sorted using samtools (version 1.3), and expression counts were generated by featureCount (version 1.5.0‐p3). Normalization and estimation of dispersion was performed by edgeR (version 3.38.1).

### Differential expression analysis and statistics

Differential gene expression analysis was performed using R2 Bioinformatics platform (http://r2.amc.nl; Amsterdam, The Netherlands) without false discovery rate correction. Differences were considered significant if *p* ≤ 0.01. The output consisted of heat maps, volcano plots, Venn diagrams, and expression box plots.

### Real‐time PCR

Real‐time PCR was used to determine gene expression of differentially expressed genes (DEGs) in RNA samples from donors used for RNA‐seq analysis, as well as in RNA samples from other donors that were not analyzed by RNA‐seq (Fig. 1*C*). cDNA synthesis was performed in a 20 μL reverse transcription reaction mixture using SuperScript IV VILO Master Mix kit (Thermo Fisher Scientific) according to the manufacturer's instructions. Real‐time PCR was performed on 10 μL reaction mixtures containing 2 μL of five times‐diluted cDNA, 1 μL of a solution containing 4 pmol/L (*DLK1*, *EGR1*, *FAF1*, *H3F3B, PAN2*, *RNF213*, *SAMD4A*, *TBC1D24*, *EGFEM1P*, *HOXD4*, *SNORD91B*, *SNX9*) or 10 pmol/L (*ADRA2B*) of forward and reverse primers, and 5 μL LightCycler 480 SYBR Green I Master (Roche Sequencing Solutions). The following primer sets were used: *ADRA2B*, forward: 5′‐GTTGTTGAGGCCAGAGTATC‐3′; reverse: 5′ CCACCTGCATGTCGCAATAAG 3′; *DLK1*, forward: 5′‐TTTCGGCCACAGCACCTATG‐3′; reverse: 5′‐CCAGGCTCACGCAGGTCCTGTT‐3′; *EGR1*: forward: 5′‐CAGCAGCAGCAGCACCTTCA‐3′; reverse: 5′‐CACAAGGTGTTGCCACTGTT‐3′; *FAF1*: forward: 5′‐GGCCAACTTCTGCTACAGAC‐3′; reverse: 5′‐GTCATCTCCATCGCTATCAC‐3′; *H3F3B*: forward: 5′‐TTCAGAGCGCAGCCATCGGT‐3′; reverse: 5′‐GCGAGCCAACTGGATGTCTT‐3′; *PAN2*: forward: 5′‐TCCTACCCTGATGGTAGCAAA‐3′; reverse: 5′‐ATGTTGCGGGTCTGAATCGTG‐3′; *RNF213*: forward: 5′‐GGCTGTCGCAGGAGTACTT‐3′; reverse: 5′‐GCCTGTGACCTCTGATTCTA‐3′; *SAMD4A*: forward: 5′‐ACCAGCGCAACACCACAGCTA‐3′; reverse: 5′‐ACCAGCGCAACACCACAGCTA‐3′; *TBC1D24*: forward: 5”‐ACTTCCGCTCGGAGATCGTC‐3′; reverse: 5′‐CCTCCTTCTGCGTGGTCTT‐3′; *EGFEM1P*: forward: 5′‐TGGCTGCACTTCAGAATGTC‐3′; reverse: 5′‐CAGGGTGCAGAGGAGAACCA‐3′; *HOXD4*: forward: 5′‐TGAAGAAGGTGCACGTGAA‐3′; reverse: 5′‐ATCTTGATCTGGCGCTCCGA‐3′; *SNORD91B*: forward: 5′‐GTCTGAACCTGTCTGAAGCATCC‐3′; reverse: 5′‐AAGCCTCAGTATCACACAGAAGT‐3′; *SNX9*: forward: 5′‐AGGCCTGGATGACCAGGATGT‐3′; reverse: 5′‐CCAAGTCAGGTGCCTCTGGTT‐3′. Real‐time PCR was performed on a LightCycler 480 Instrument II (Roche Sequencing Solutions): 10 minutes at 95°C, 45 cycles each composed of 10 seconds at 95°C, 5 seconds at 60°C (for *COX‐2*, 56°C), 10 seconds at 72°C, and 5 seconds at 78°C. Relative gene expression was calculated by the 2_Ct(housekeeping gene)–Ct(target gene)_ method. TATA binding protein gene (*TBP*: forward: 5′‐AGTTCTGGGATTGTACCGCA‐3′; reverse: 5′‐TCCTCATGATTACCGCAGCA‐3′) was used as housekeeping gene to normalize for the amount of total RNA per sample.

## Statistical analysis

DESeq2 was used to analyze differential gene expression between unloaded and 2000 or 8000 μɛ loaded bone. Differences were considered significant if *p* < 0.01. One‐way ANOVA was used to test differences in gene expression of *DLK1*, *ADRA2B*, and *H3F3B* measured by RNA‐seq between unloaded and 2000 or 8000 μɛ loaded bone at 0 or 6 hours post‐culture. Differences were considered significant if *p* < 0.05. One‐way ANOVA (mixed‐effect analysis) was used to test differences in gene expression of *EGR1*, *FAF1*, *PAN2*, *RNF213*, *SAMD4A*, *TBC1D24*, *EGFEM1P*, *HOXD4*, *SNORD91B*, and *SNX9* measured by RNA‐seq between unloaded and 2000 or 8000 μɛ loaded bone at 6 or 24 hours post‐culture. Differences were considered significant if *p* < 0.05. One‐way ANOVA (mixed‐effect analysis) was also used to test differences in gene expression of *ADRA2B*, *EGR1*, *FAF1*, *H3F3B*, *PAN2*, *RNF213*, *SAMD4A*, *TBC1D24*, *EGFEM1P*, *HOXD4*, *SNORD91B*, and *SNX9* measured by real‐time PCR between unloaded, 2000 μɛ loaded, and 8000 μɛ loaded bone at 0, 6, and 24 hours post‐culture. Differences were considered significant if *p* < 0.05. Two‐way ANOVA (mixed‐effect analysis) was used to determine whether DEGs were differentially expressed between different post‐culture time points (0, 6, 24 hours) at different loading magnitudes (0, 2000, 8000 μɛ). Differences were considered significant if *p* < 0.05. All analyses were performed using GraphPad Prism software 9 (GraphPad, San Diego, CA, USA).

## Results

### Total number of DEGs

The total number of DEGs between unloaded and 2000 μɛ loaded bone or between unloaded and 8000 μɛ loaded bone at 0, 6, and 24 hours post‐culture was 1495 (Table S1). Practical considerations made us first study the genes that were differentially expressed between both unloaded and 2000 μɛ loaded bone *and* between unloaded and 8000 μɛ loaded bone, because these genes might be most relevant for the osteocyte response to mechanical loading.

### DEGs (0 hours post‐culture)

#### RNA‐seq

**Fig. 2:**
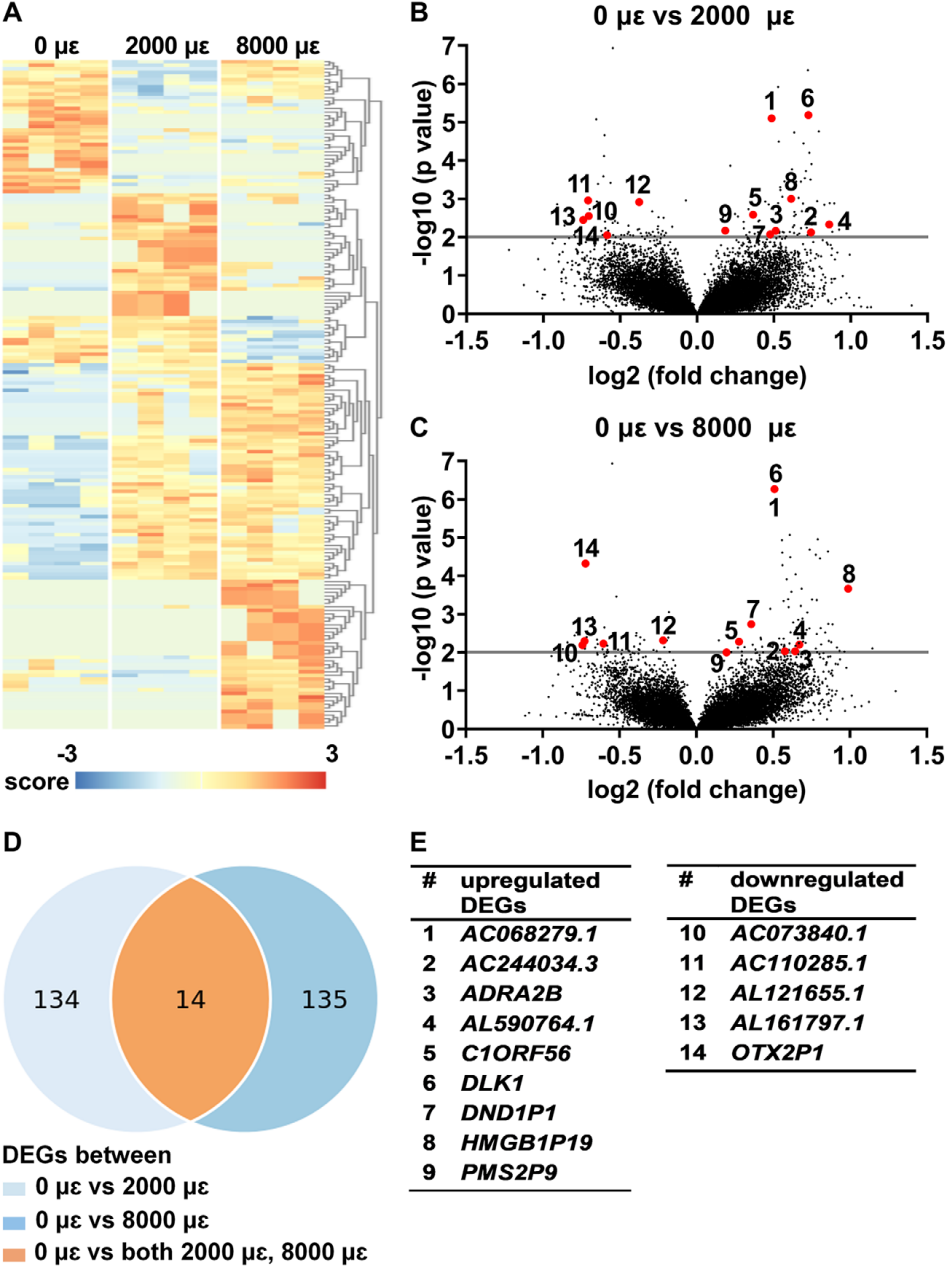
Differential expression analysis between unloaded (0 μɛ), 2000 μɛ, and 8000 μɛ loaded human cortical bone from four donors without post‐culture using RNA‐seq. (*A*) Heat map: all DEGs. Score: relative gene expression. (*B*) Volcano plot: DEGs between unloaded (0 μɛ) and 2000 μɛ loaded bone. Red dots: numbers corresponding to genes listed in *E*. (*C*) Volcano plot: DEGs between unloaded (0 μɛ) and 8000 μɛ loaded bone. Red dots: numbers corresponding to genes listed in *E*. (*D*). Venn diagram: 14 shared DEGs between unloaded (0 μɛ) and 2000 μɛ loaded bone, and between unloaded (0 μɛ) and 8000 μɛ loaded bone. (*E*) List of 14 upregulated or downregulated shared DEGs between unloaded (0 μɛ) and 2000 μɛ loaded bone, and between unloaded (0 μɛ) and 8000 μɛ loaded bone. *n* = 4.

Differential gene expression analysis was performed to study the effect of mechanical loading at 2000 or 8000 μɛ on the transcriptome of osteocytes in their native matrix without post‐culture. Transcriptional differences were observed between unloaded bone (0 μɛ), bone loaded at 2000 μɛ, and bone loaded at 8000 μɛ without post‐culture (Fig. 2*A*). We found 148 DEGS between unloaded and 2000 μɛ loaded bone (Fig. 2*B*,*D*), and 149 between unloaded and 8000 μɛ loaded bone (Fig. 2*C*,*D*). DEGs between unloaded and 2000 μɛ loaded bone or between unloaded and 8000 μɛ loaded bone without post‐culture were analyzed using the Database for Annotation, Visualization, and Integrated Discovery (DAVID) Bioinformatics Resources online tools, producing a list of Kyoto Encyclopedia of Genes and Genomes (KEGG) pathways (Table S2). Fourteen genes were differentially expressed between unloaded and 2000 μɛ loaded bone, as well as between unloaded and 8000 μɛ loaded bone (Fig. 2*B*–*D*). Nine of these 14 DEGs were upregulated, ie, *AC068279.1* (novel pseudogene; ENSG00000224881), *AC244034.3* (novel transcript, sense intronic to RASSF5; ENSG00000279946), adrenoceptor alpha 2B (*ADRA2B*; ENSG00000274286), *AL590764.1* (novel transcript; ENSG00000228427), chromosome 1 open reading frame 56 (*C1orf56*; *ENSG00000143443*), delta‐like non‐canonical ligand 1 (*DLK1*; ENSG00000185559), DND microRNA‐mediated repression inhibitor 1 (*DND1P1*, pseudogene 1; ENSG00000264070), high mobility group box 1 pseudogene 19 (*HMGB1P19*; ENSG00000253463), and PMS1 homolog 2, mismatch repair system component pseudogene 9 (*PMS2P9*; ENSG00000233448) and five DEGS were downregulated, ie, *AC073840.1* (novel transcript; ENSG00000286035), *AC110285.1* (novel transcript; ENSG00000262223), *AL121655.1* (novel transcript, antisense to DPY30; ENSG00000271228), *AL161797.1* (novel transcript; ENSG00000287015), and OTX2 pseudogene 1 (*OTX2P1*; ENSG00000234644) (Fig. 2*E*).

**Fig. 3:**
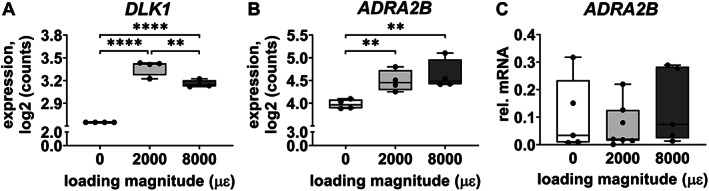
Gene expression of two of the shared 14 DEGs, related to bone metabolism, in osteocytes in unloaded (0 μɛ), 2000 μɛ, and 8000 μɛ loaded human cortical bone without post‐culture. (*A*) *DLK1* gene expression (RNA‐seq; *n* = 4). (*B*) *ADRA2B* gene expression (RNA‐seq; *n* = 4). (*C*) *ADRA2B* gene expression (real‐time PCR; 0 μɛ, *n* = 5; 2000 μɛ, *n* = 7; 8000 μɛ, *n* = 5). **p < 0.01, ****p < 0.0001.

Gene expression of *DLK1* and *ADRA2B* was analyzed because they are related to bone metabolism. Mechanical loading at both 2000 and 8000 μɛ without post‐culture increased *DLK1* expression compared to static condition (*p* < 0.0001; Fig. 3*A*). Mechanical loading at 2000 μɛ (*p* = 0.0070) and 8000 μɛ (*p* = 0.0022) increased *ADRA2B* expression compared to static condition without post‐culture (Fig. 3*B*).

RNA‐seq data on mechanosensitive gene expression of *SOST*, *COX‐2*, and *MEPE* was not significantly different between unloaded bone and 2000 or 8000 μɛ loaded bone without post‐culture (Fig. S1).

#### Real‐time PCR

*DLK1* gene expression was not detectable in unloaded bone, nor in 2000 or 8000 μɛ loaded bone without post‐culture. *ADRA2B* gene expression was not significantly different between unloaded bone and 2000 or 8000 μɛ loaded bone without post‐culture (Fig. 3*C*).

Real‐time PCR data on mechanosensitive gene expression of *SOST*, *COX‐2*, and *MEPE* was not significantly different between unloaded bone and 2000 or 8000 μɛ loaded bone without post‐culture (Fig. S2).

### DEGs (6 hours post‐culture)

#### RNA‐seq

**Fig. 4:**
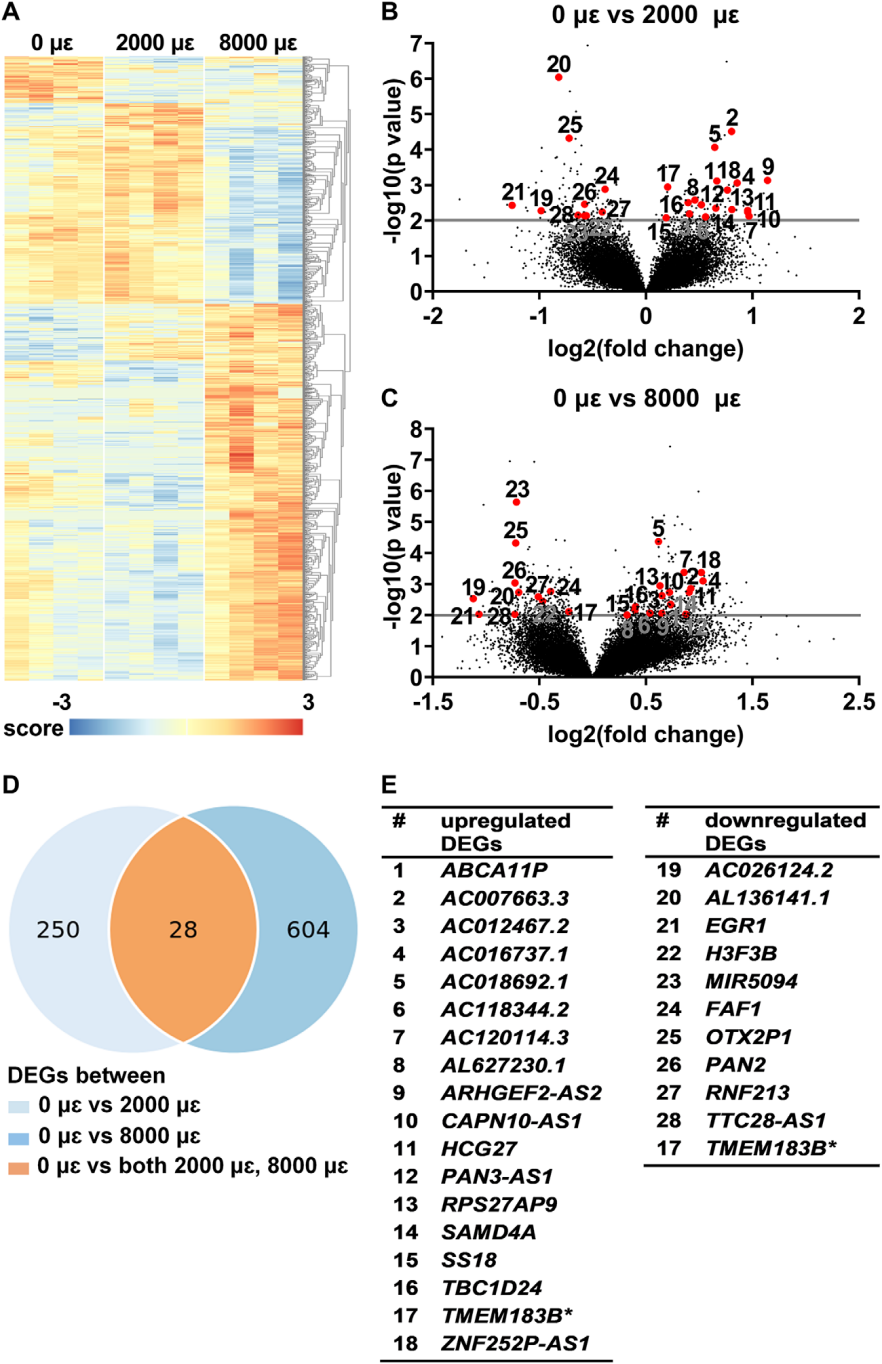
Differential expression analysis between unloaded (0 μɛ), 2000 μɛ, and 8000 μɛ loaded human cortical bone from five donors with 6 hours post‐culture using RNA‐seq. (*A*) Heat map: all DEGs. Score: relative gene expression. (*B*) Volcano plot: DEGs between unloaded (0 μɛ) and 2000 μɛ loaded bone. Red dots: numbers corresponding to genes listed in *E*. (*C*) Volcano plot: DEGs between unloaded (0 μɛ) and 8000 μɛ loaded bone. Red dots: numbers corresponding to genes listed in *E*. (*D*) Venn diagram: 28 shared DEGs between unloaded (0 μɛ) and 2000 μɛ loaded bone, and between unloaded (0 μɛ) and 8000 μɛ loaded bone. (*E*) List of 28 upregulated or downregulated shared DEGs between unloaded (0 μɛ) and 2000 μɛ loaded bone, and between unloaded (0 μɛ) and 8000 μɛ loaded bone. *TMEM183B gene expression was upregulated in 2000 μɛ loaded bone, but downregulated in 8000 μɛ loaded bone compared to unloaded bone. *n* = 4.

Differential gene expression analysis was performed to study the effect of mechanical loading at 2000 or 8000 μɛ on the transcriptome of osteocytes in their native matrix after 6 hours post‐culture. Transcriptional differences were observed between unloaded bone (0 μɛ), bone loaded at 2000 μɛ, and bone loaded at 8000 μɛ with 6 hours post‐culture (Fig. 4*A*). We found 278 DEGs between unloaded and 2000 μɛ loaded bone (Fig. 4*B*,*D*), and 632 between unloaded and 8000 μɛ loaded bone (Fig. 4*C*,*D*). DEGs between unloaded and 2000 μɛ loaded bone or between unloaded and 8000 μɛ loaded bone with 6 hours post‐culture were analyzed using DAVID Bioinformatics Resources online tools, producing a list of KEGG pathways (Table S3). Twenty‐eight genes were differentially expressed between unloaded and 2000 μɛ loaded bone, as well as between unloaded and 8000 μɛ loaded bone (Fig. 4*B*–*D*). Eighteen of these 28 DEGs were upregulated, ie, ATP binding cassette subfamily A member 11, pseudogene (*ABCA11P*; ENSG00000251595), *AC007663.3* (novel transcript; ENSG00000273139), *AC012467.2* (novel transcript, antisense to IL17RB; ENSG00000271976), *AC016737.1* (novel transcript; ENSG00000271151), *AC018692.1* (novel transcript; ENSG00000279321), *AC118344.2* (to be experimentally confirmed; ENSG00000279759), *AC120114.3* (novel transcript, sense intronic to KCTD13; ENSG00000279789), *AL627230.1* (family with sequence similarity 27 member pseudogene; ENSG00000275493), ARHGEF2 antisense RNA 2 (*ARHGEF2‐AS2*; ENSG00000273002), CAPN10 divergent transcript (*CAPN10‐AS1*; ENSG00000260942), HLA complex group 27 (*HCG27*; ENSG00000206344), PAN3 antisense RNA 1 (*PAN3‐AS1*; ENSG00000261485), RPS27A pseudogene 9 (*RPS27AP*; ENSG00000242706), sterile alpha motif domain containing 4A (*SAMD4A*; ENSG00000020577), SS18 subunit of BAF chromatin remodeling complex (*SS18*; ENSG00000141380), TBC1 domain family member 24 (*TBC1D24*; ENSG00000162065), transmembrane protein 183B, pseudogene (*TMEM183B*; ENSG00000224831), and ZNF252P antisense RNA 1 (*ZNF252P‐AS1*; ENSG00000255559), and 11 DEGs were downregulated, ie, *AC026124.2* (novel transcript, sense intronic to LEMD3; ENSG00000276853), *AL136141.1* (novel transcript; ENSG00000270755), early growth response 1 (*EGR1*; ENSG00000120738), H3.3 histone B (*H3F3B*; ENSG00000132475), microRNA 5094 (*MIR5094*; ENSG00000264966), Fas associated factor 1 (*FAF1*; ENSG00000185104), OTX2 pseudogene 1 (*OTX2P1*; ENSG00000234644), poly(A) specific ribonuclease subunit PAN2 (*PAN2*; ENSG00000135473), ring finger protein 213 (*RNF213*; ENSG00000173821), TTC28 antisense RNA 1 (*TTC28‐AS1*; ENSG00000235954), and transmembrane protein 183B, pseudogene (*TMEM183B*; ENSG00000224831) (Fig. 4*E*). *TMEM183B* gene expression was upregulated in bone loaded at 2000 μɛ compared to unloaded bone, but downregulated in bone loaded at 8000 μɛ compared to unloaded bone (Fig. 4*E*).

**Fig. 5:**
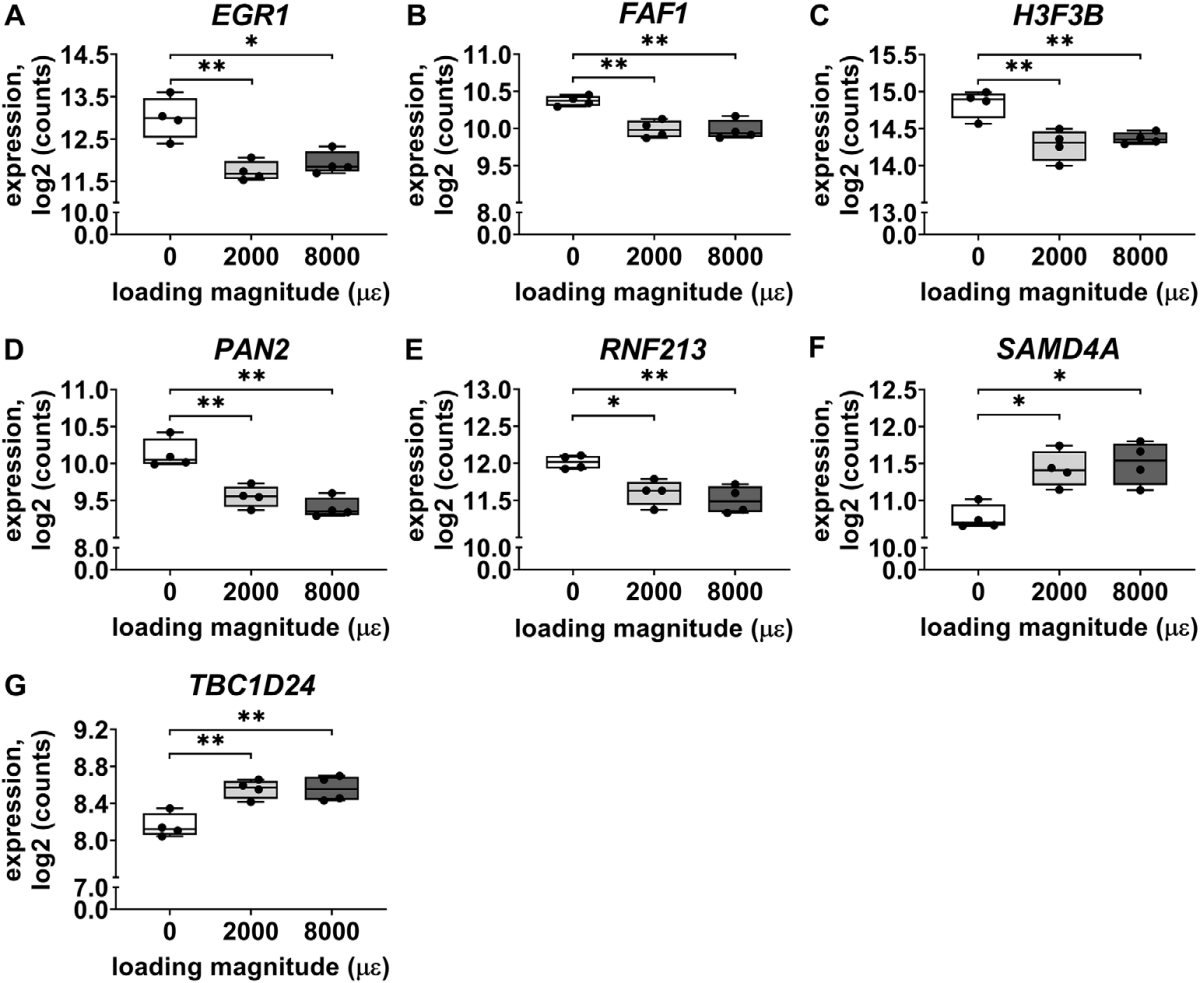
RNA‐seq‐obtained gene expression of seven of shared 28 DEGs, related to bone metabolism, in osteocytes in unloaded (0 μɛ), 2000 μɛ, and 8000 μɛ loaded human cortical bone with 6 hours post‐culture. (*A*) *EGR1* gene expression. (*B*) *FAF1* gene expression. (*C*) *H3F3B* gene expression. (*D*) *PAN2* gene expression. (*E*). *RNF213* gene expression. (*F*) *SAMD4A* gene expression. (*G*) *TBC1D24* gene expression. *n* = 4. *p < 0.05, **p < 0.01.

Gene expression of *EGR1*, *FAF1*, *H3F3B*, *PAN2*, *RNF213*, *SAMD4A*, and *TBC1D24* was analyzed since they are related to bone metabolism. Mechanical loading at both 2000 and 8000 μɛ with 6 hours post‐culture decreased *EGR1* (*p* = 0.0067, 0.0137), *FAF1* (*p* = 0.0032, 0.0020), *H3F3B* (*p* = 0.0029, 0.0086), *PAN2* (*p* = 0.0092, 0.0032), and *RNF213* (*p* = 0.0137, 0.033) gene expression compared to static condition (Fig. 5*A*–*E*). It increased *SAMD4A* (*p* = 0.0208, 0.0117) and *TBC1D24* (*p* = 0.0038, 0.0034) compared to static condition (Fig. 5*F*,*G*).

RNA‐seq data on mechanosensitive gene expression of *SOST*, *COX‐2*, and *MEPE* was not significantly different between unloaded bone and 2000 or 8000 μɛ loaded bone with 6 hours post‐culture (Fig. S1).

#### Real‐time PCR

**Fig. 6:**
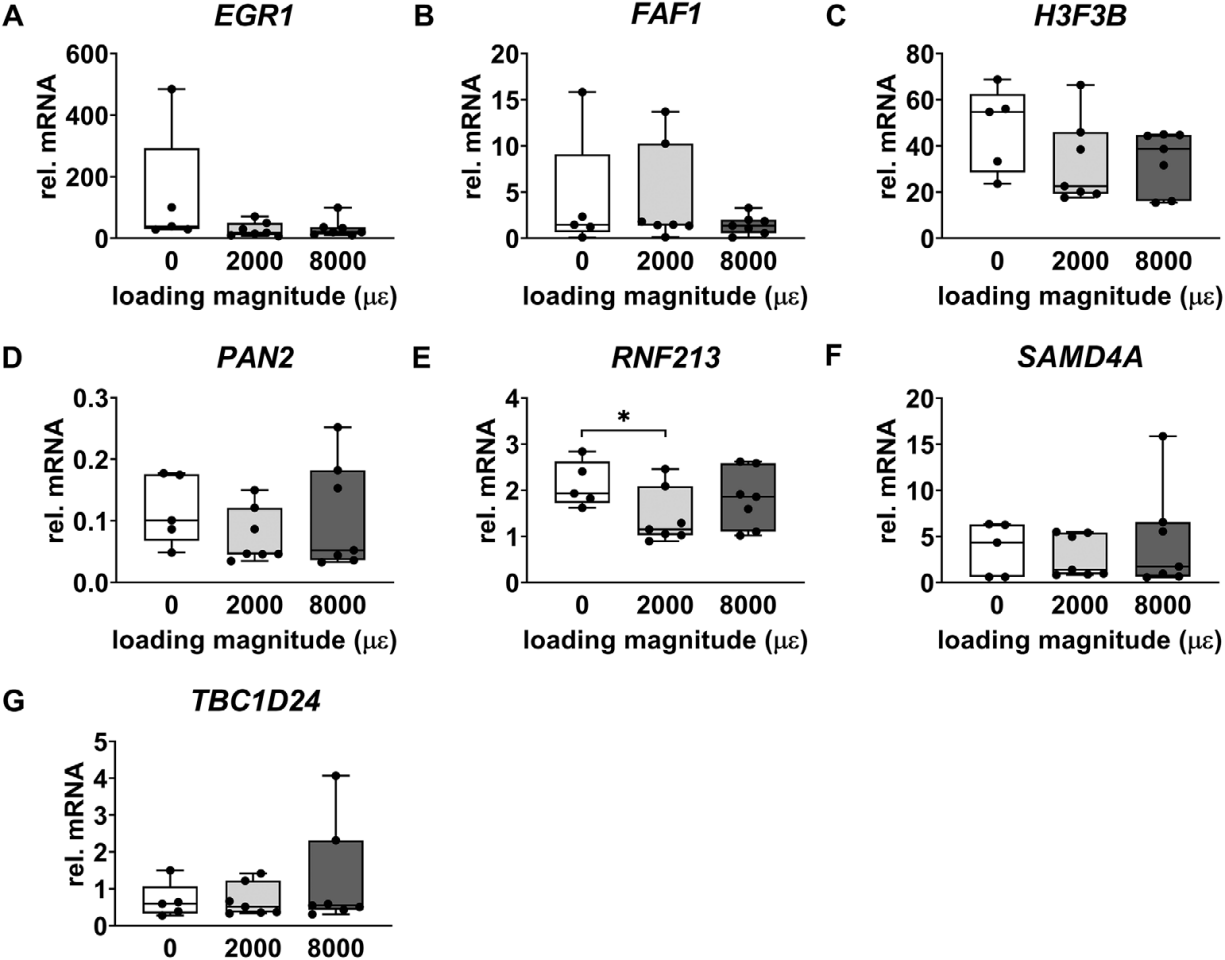
Real‐time PCR‐obtained gene expression of seven of the shared 28 DEGs, related to bone metabolism, in osteocytes in unloaded (0 μɛ), 2000 μɛ, and 8000 μɛ loaded human cortical bone with 6 hours post‐culture. (*A*) *EGR1* gene expression. (*B*) *FAF1* gene expression. (*C*) *H3F3B* gene expression. (*D*) *PAN2* gene expression. (*E*) *RNF213* gene expression. (*F*) *SAMD4A* gene expression. (*G*) *TBC1D24* gene expression. 0 μɛ, *n* = 5; 2000 μɛ, *n* = 7; 8000 μɛ, *n* = 7. *p < 0.05.

Gene expression of *EGR1*, *FAF1*, *H3F3B*, *PAN2*, *SAMD4A*, and *TBC1D24* was not significantly different between unloaded bone and 2000 or 8000 μɛ loaded bone with 6 hours post‐culture (Fig. 6*A*–*D*,*F*,*G*). *RNF213* gene expression was decreased by mechanical loading at 2000 μɛ compared to static condition with 6 hours post‐culture (*p* = 0.0213) (Fig. 6*E*), which was consistent with the RNA‐seq results.

Real‐time PCR data on mechanosensitive gene expression of *SOST*, *COX‐2*, and *MEPE* was not significantly different between unloaded bone and 2000 or 8000 μɛ loaded bone with 6 hours post‐culture (Fig. S2).

### DEGs (24 hours post‐culture)

#### RNA‐seq

**Fig. 7:**
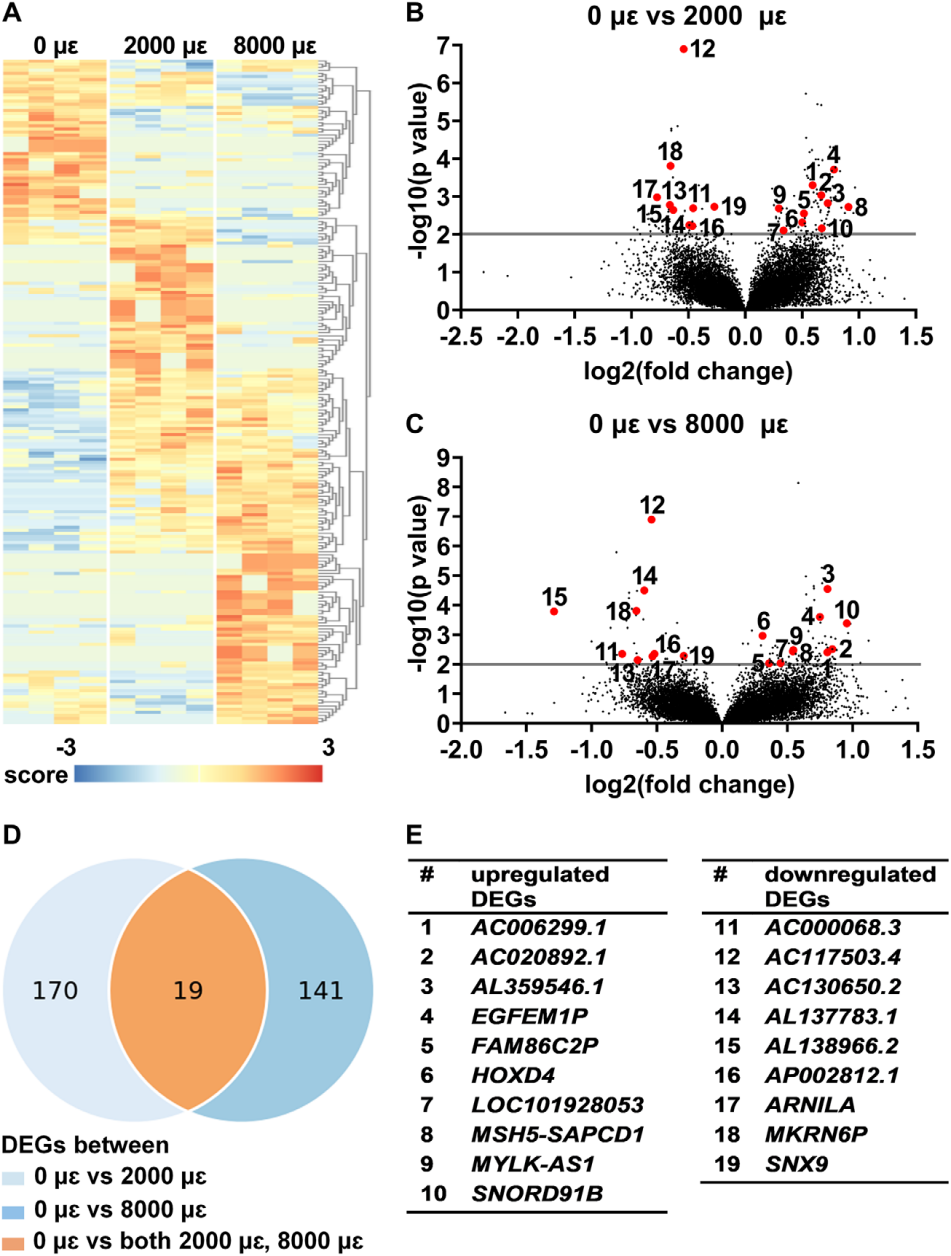
Differential expression analysis between unloaded (0 μɛ), 2000 μɛ, and 8000 μɛ loaded human cortical bone from five donors with 24 hours post‐culture using RNA‐seq. (*A*) Heat map: all DEGs. Score: relative gene expression. (*B*) Volcano plot: DEGs between unloaded (0 μɛ) and 2000 μɛ loaded bone. Red dots: numbers corresponding to genes listed in *E*. (*C*) Volcano plot: DEGs between unloaded (0 μɛ) and 8000 μɛ loaded bone. Red dots: numbers corresponding to genes listed in *E*. (*D*) Venn diagram: 19 shared DEGs between unloaded (0 μɛ) and 2000 μɛ loaded bone, and between unloaded (0 μɛ) and 8000 μɛ loaded bone. (*E*) List of 19 upregulated or downregulated shared DEGs between unloaded (0 μɛ) and 2000 μɛ loaded bone, and between unloaded (0 μɛ) and 8000 μɛ loaded bone. *n* = 4.

Differential gene expression analysis was performed to study the effect of mechanical loading at 2000 or 8000 μɛ on the transcriptome of osteocytes in their native matrix after 24 hours post‐culture. Transcriptional differences were observed between unloaded bone (0 μɛ), bone loaded at 2000 μɛ, and bone loaded at 8000 μɛ with 24 hours post‐culture (Fig. 7*A*). We found 189 DEGs between unloaded and 2000 μɛ loaded bone (Fig. 7*B*,*D*), and 160 between unloaded and 8000 μɛ loaded bone (Fig. 7*C*,*D*). DEGs between unloaded and 2000 μɛ loaded bone or between unloaded and 8000 μɛ loaded bone with 24 hours post‐culture were analyzed using DAVID Bioinformatics Resources online tools, producing a list of KEGG pathways (Table S4). Nineteen genes were differentially expressed between unloaded and 2000 μɛ loaded bone, as well as between unloaded and 8000 μɛ loaded bone (Fig. 7*B*–*D*). Ten of these 19 DEGs were upregulated, ie, *AC006299*.1 (novel transcript, antisense to SVIP; ENSG00000246225), AC020892*.1* (novel transcript; ENSG00000259241), *AL359546.1* (novel transcript; ENSG00000287564), EGF like and EMI domain containing 1, pseudogene (*EGFEM1P*; ENSG00000206120), family with sequence similarity 86 member C2, pseudogene (*FAM86C2P*; ENSG00000160172), homeobox D4 (*HOXD4*; ENSG00000170166), *LOC101928053* (novel transcript, antisense to ZBED5; ENSG00000246308), MSH5‐SAPCD1 readthrough (NMD candidate) (*MSH5‐SAPCD1*; ENSG00000255152), MYLK antisense RNA 1 (*MYLK‐AS1*; ENSG00000239523), and small nucleolar RNA, C/D box 91B (*SNORD91B*; ENSG00000275084), and nine DEGs were downregulated, ie, *AC000068.3* (novel transcript, antisense to UFD1L; ENSG00000273300), *AC117503.4* (novel transcript; ENSG00000280300), *AC130650.2* (novel transcript; ENSG00000276564), *AL137783.1* (to be experimentally confirmed; ENSG00000279960), *AL138966.2* (novel transcript, antisense to ring finger protein (C3H2C3 type) 6; ENSG00000277368), *AP002812.1* (ribosomal protein L21 (RPL21) pseudogene; ENSG00000241782), androgen receptor negatively regulated lncRNA (*ARNILA*; ENSG00000235072), makorin ring finger protein 6, pseudogene (*MKRN6P*; ENSG00000227154), and sorting nexin 9 (*SNX9*; ENSG00000130340) (Fig. 7*E*).

**Fig. 8:**
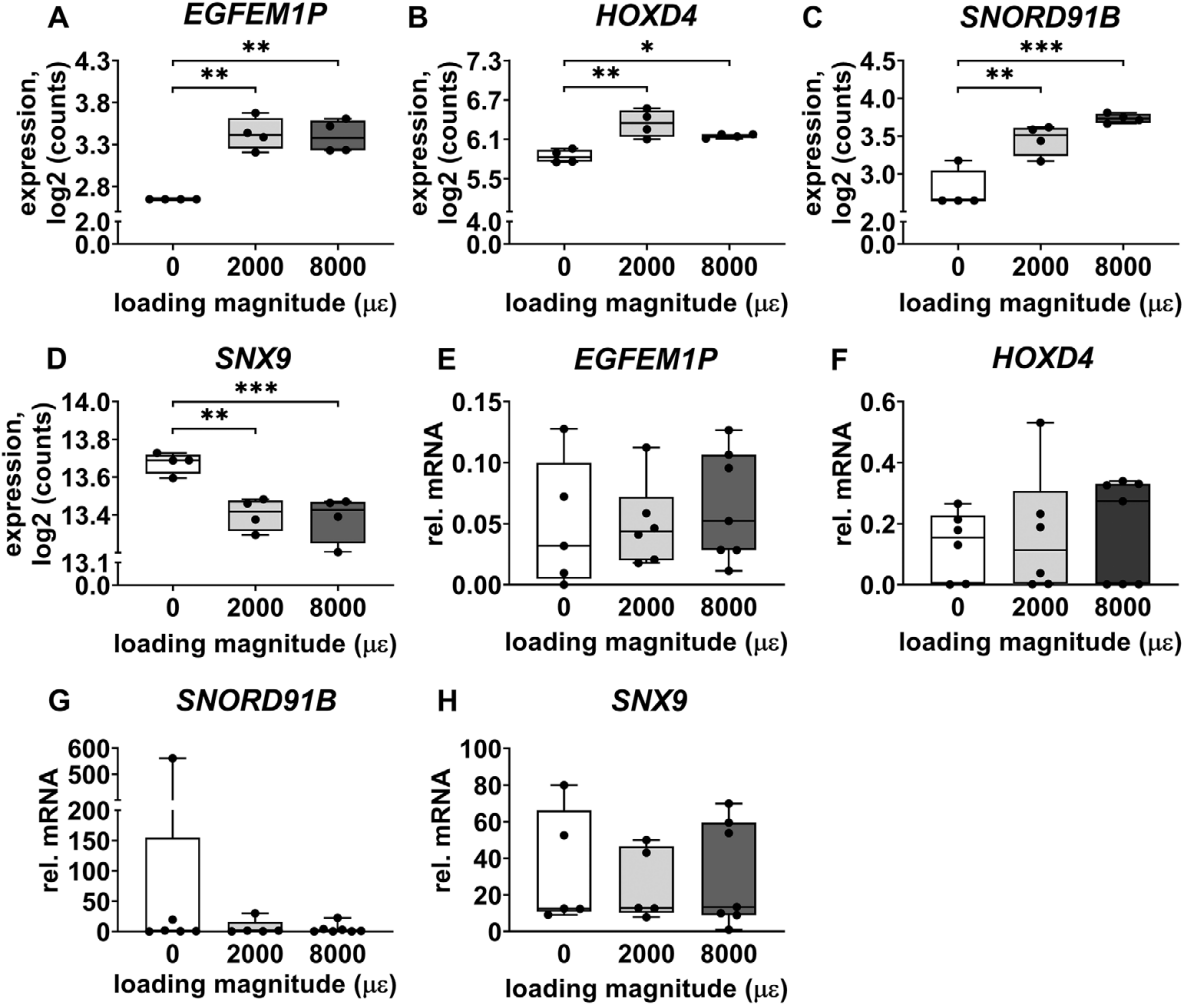
Gene expression of four of 19 DEGs, related to bone metabolism, in osteocytes in unloaded (0 μɛ), 2000 μɛ, and 8000 μɛ loaded human cortical bone with 24 hours post‐culture. (*A*) *EGFEM1P* gene expression (RNA‐seq; *n* = 4). (*B*) *HOXD4* gene expression (RNA‐seq; *n* = 4). (*C*) *SNORD91B* gene expression (RNA‐seq; *n* = 4). (*D*) *SNX9* gene expression (RNA‐seq; *n* = 4). (*E*) *EGFEM1P* gene expression (real‐time PCR; 0 μɛ, *n* = 5; 2000 μɛ, *n* = 6; 8000 μɛ, *n* = 7). (*F*) *HOXD4* gene expression (real‐time PCR; 0 μɛ, *n* = 5; 2000 μɛ, *n* = 6; 8000 μɛ, *n* = 7). (*G*) *SNORD91B* gene expression (real‐time PCR; 0 μɛ, *n* = 5; 2000 μɛ, *n* = 6; 8000 μɛ, *n* = 7). (*H*) *SNX9* gene expression (real‐time PCR; 0 μɛ, *n* = 5; 2000 μɛ, *n* = 6; 8000 μɛ, *n* = 7). *p < 0.05, **p < 0.01, ***p < 0.005.

Gene expression of *EGFEM1P*, *HOXD4*, *SNORD91B*, and *SNX9* was analyzed because they are related to bone metabolism. Mechanical loading at both 2000 and 8000 μɛ with 24 hours post‐culture increased gene expression of *EGFEM1P* (*p* = 0.0018, 0.0019), *HOXD4* (*p* = 0.0039, 0.0237), and *SNORD91B* (*p* = 0.0024, 0.0002) compared to static condition, whereas it decreased *SNX9* expression (*p* = 0.0034, 0.0008; Fig. 8*A*–*D*).

RNA‐seq data on mechanosensitive gene expression of *SOST*, *COX‐2*, and *MEPE* was not significantly different between unloaded bone and 2000 or 8000 μɛ loaded bone with 24 hours post‐culture (Fig. S1).

#### Real‐time PCR

Gene expression of *EGFEM1P*, *HOXD4*, *SNORD91B*, and *SNX9* was not significantly different between unloaded bone and 2000 or 8000 μɛ loaded bone with 24 hours post‐culture (Fig. 8*E‐H*).

Real‐time PCR data on mechanosensitive gene expression of *SOST*, *COX‐2*, and *MEPE* was not significantly different between unloaded bone and 2000 or 8000 μɛ loaded bone with 24 hours post‐culture (Fig. S2).

### Gene expression at 0, 6, and 24 hours post‐culture

**Fig. 9:**
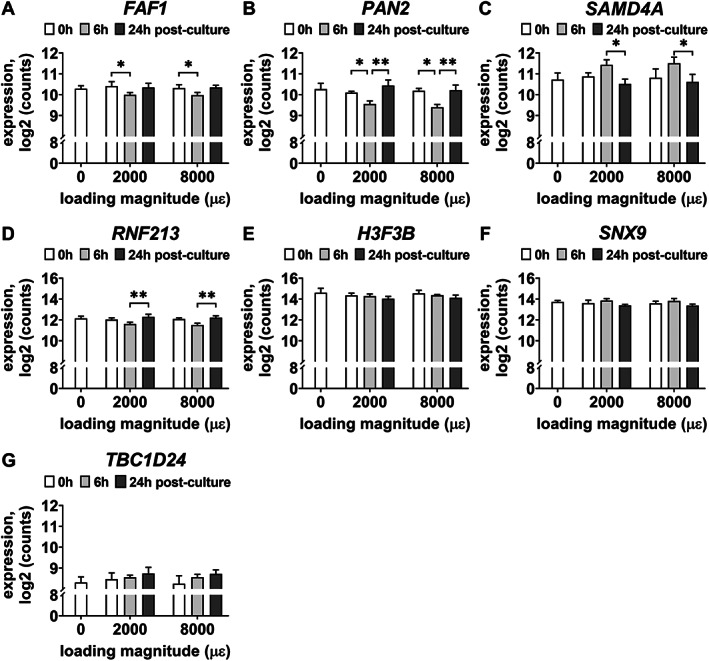
RNA‐seq‐obtained gene expression of DEGs in osteocytes in their native bone matrix in response to mechanical loading at 2000 and 8000 μɛ at different post‐culture times (0, 6, and 24 hours). (*A*) *FAF1* gene expression. (*B*) *PAN2* gene expression. (*C*) *SAMD4A* gene expression. (*D*) *RNF213* gene expression. (*E*) *H3F3B* gene expression. (*F*) *SNX9* gene expression. (*G*) *TBC1D24* gene expression. 0 μɛ, *n* = 6; 2000 μɛ, *n* = 4; 8000 μɛ, *n* = 4. *p < 0.05, **p < 0.01.

To study the effect of post‐culture time on gene expression of DEGs, the RNA‐seq gene expression results at 0, 6, and 24 hours post‐culture of either not loaded or mechanically loaded bone explants (2000 or 8000 μɛ) was analyzed. Seven DEGs were differentially expressed between the different post‐culture times (Fig. 9). *FAF1* expression was decreased at 6 hours post‐culture compared to 0 hours post‐culture by mechanical loading at 2000 and 8000 μɛ (*p* = 0.0287; Fig. 9*A*)*. PAN2* expression was decreased at 6 hours post‐culture compared to 0 hours post‐culture by mechanical loading at 2000 and 8000 μɛ (*p* = 0.0339), but increased at 24 hours post‐culture compared to 6 hours post‐culture by mechanical loading at 2000 and 8000 μɛ (*p* = 0.0069; Fig. 9*B*)*. SAMD4A* expression was decreased at 24 hours post‐culture compared to 6 hours post‐culture by mechanical loading at 2000 and 8000 μɛ (*p* = 0.0481; Fig. 9*C*)*. RNF213* expression was increased at 24 hours post‐culture compared to 6 hours post‐culture by mechanical loading at 2000 and 8000 μɛ (*p* = 0.0074; Fig. 9*D*). Gene expression of *H3F3B* (*p* = 0.0141), *SNX9* (*p* = 0.0062), and *TBC1D24* (*p* = 0.0158) was affected by post‐culture time (0, 6, and 24 hours) according to two‐way analysis of variance (ANOVA) (mixed‐effect analysis), but gene expression of *H3F3B*, *SNX9*, and *TBC1D24* was not significantly different between 0 and 6 hours post‐culture, between 0 and 24 hours post‐culture, and between 6 and 24 hours post‐culture after mechanical loading at 2000 and 8000 μɛ (Fig. 9*E*–*G*).

## Discussion

This study mapped the transcriptome of human osteocytes in their native matrix in response to mechanical loading using RNA‐seq. Forty‐seven genes were differentially expressed between unloaded and 2000 or 8000 μɛ loaded osteocytes at 6 or 24 hours post‐culture without mechanical loading. Eleven of the 47 DEGs were related to bone metabolism, and might be mechanosensitive genes. One of the genes was *RNF213*, that might play a role in mechanical adaptation of bone by regulating angiogenesis, which is a prerequisite for successful bone formation. Expression analysis of the 11 DEGs in metabolic bone diseases might help to better understand the pathogenesis of these diseases and thereby optimize clinical treatment. Further functional studies of these genes are needed to assess whether they are related to known mechanotransduction pathways in osteocytes, thereby contributing to a more complete view of how osteocytes orchestrate bone adaptation to mechanical loading.

Physiological loading of whole bone reaches up to 2000 to 3000 μɛ during strenuous activity.^(^ ^49^, ^50^ ^)^ Therefore, 2000 μɛ in this study mimicked physiological loading. Strains above 3000 μɛ induce pathological overload on bone, leading to woven bone formation instead of lamellar bone formation.^(^ ^51^ ^)^ Strains above 25,000 μɛ cause bone fracture in young adult mammals.^(^ ^51^ ^)^ In our study, 8000 μɛ mimicked pathological overloading. This loading magnitude was below the fracture threshold.^(^ ^48^ ^)^

We found that 14 genes were differentially expressed between unloaded bone and 2000 and 8000 μɛ loaded bone without post‐culture after mechanical loading. Gene transcription in mammalian cells takes 1 minute/kilobase pair (kbp).^(^ ^53^ ^)^ To transcribe one gene of average size (10 kbp) takes around 10 minutes.^(^ ^53^ ^)^ In our study, for practical reasons, there was a ~10‐minute time period between mechanical loading and freezing in liquid nitrogen. Therefore, there was ample time to allow gene transcription in the bone explants without post‐culture after mechanical loading, especially for small genes such as ADRA2B (only 3.7 kbp). We found that *ADRA2B* and *DLK1* were differentially expressed between unloaded bone and 2000 and 8000 μɛ loaded bone without post‐culture, indicating that these genes are involved in the early response of osteocytes to mechanical loading. Superficial cells (eg, osteoblasts, bone lining cells, osteoclasts) were removed by collagenase type II incubation. It has been reported that RNA isolated from mouse cortical bone is mainly from osteocytes.^(^ ^54^ ^)^ Therefore, expression of genes related to bone metabolism detected by RNA‐seq in our study was most likely from osteocytes. The *ADRA2B* gene encodes for the α‐2B‐adrenergic receptor, which is a subtype of α‐2 adrenal receptors. In the skeleton, α‐2 adrenal receptors mediate the sympathetic nervous system, that controls bone remodeling.^(^ ^55^ ^)^ α‐2‐Adrenoceptor knockout increases bone formation and decreases bone resorption in mice.^(^ ^55^ ^)^ Moreover, α‐2B‐adrenergic receptor expression in femoral osteocytes, osteoblasts, and osteoclasts is downregulated in α‐2A/α‐2C knockout mice.^(^ ^55^ ^)^ The α‐2B‐adrenergic receptor is associated with angiogenesis during bone regeneration on a microrough titanium implant surface.^(^ ^56^ ^)^ Taken together, our results showing that osteocytes upregulated *ADRA2B* gene expression in response to mechanical loading might indicate that osteocytes regulate bone remodeling via sympathetic signaling and angiogenesis. DLK1 stimulates the production of proinflammatory cytokines resulting in inhibition of osteoblast differentiation in humans.^(^ ^57^ ^)^ DLK1 inhibits bone formation and promotes bone resorption in mice.^(^ ^58^ ^)^ We were unable to detect *DLK1* gene expression in the bone explants using real‐time PCR. *DLK1* is mostly expressed in mesenchymal stem cells, osteoprogenitors, and osteoblasts.^(^ ^58^ ^)^ We expected low *DLK1* expression in our cortical bone explants, because most cells in these explants were osteocytes. RNA‐seq, but not real‐time PCR, detects low mRNA levels, which might explain why differential *DLK1* gene expression was detected by RNA‐seq analysis. *DLK1* might also be involved in the mechanoresponse of osteocytes affecting osteoblast differentiation and cytokine production, which inhibits bone resorption. Whether the other DEGs (*AC068279.1*, *AC244034.3*, *AL590764.1*, *C1ORF56*, *DND1P1*, *HMGB1P19*, *PMS2P9*, *AC073840.1*, *AC110285.1*, *AL121655.1*, *AL161797.1*, and *OTX2P1*), which are not related to bone metabolism, are biologically relevant is currently unknown.

At 6 hours post‐culture after mechanical loading of bone explants, DEGs related to cell metabolism were *EGR1*, *FAF1*, *H3F3B*, *PAN2*, *RNF213*, *SAMD4A*, and *TBC1D24*. EGR1 regulates the production of angiogenic and osteoclastogenic factors, thereby affecting the cell microenvironment in prostate cancer.^(^ ^59^ ^)^ EGR1 stimulates chondrocyte apoptosis, and promotes mineralization of the extracellular matrix of chondrocytes in mice.^(^ ^60^ ^)^ EGR1 regulates the accumulation of extracellular matrix of chondrocytes in humans.^(^ ^60^ ^)^ FAF1 negatively regulates the Wnt signaling pathway by inhibiting osteoblast differentiation and Wnt‐induced β‐catenin accumulation.^(^ ^61^ ^)^ *H3F3B* mutations are exclusively found in chondroblastoma, which is a chondroid matrix‐producing neoplasm.^(^ ^62^ ^)^ *PAN2* plays a role in RNA degradation of osteocytes in response to high magneto‐gravitational environment, which is regulated by connexin hemichannels.^(^ ^63^ ^)^ *RNF213* knockdown in zebrafish causes abnormal vessel sprouting in the head region, indicating that *RNF213* is involved in intracranial angiogenesis.^(^ ^64^ ^)^ *RNF213* regulates vascular development through decreasing the production of transforming growth factor‐β1, which promotes angiogenesis.^(^ ^65^ ^)^ *SAMD4A* is highly expressed in the oral cancer cell line HSC3‐C13.^(^ ^66^ ^)^ Conditioned medium obtained from HSC3‐13 cultures strongly stimulates *RANKL* expression in the mouse stromal cell line ST2 suggesting that *SAMD4A* is involved in the regulation of osteoclastic bone resorption.^(^ ^66^ ^)^ *TBC1D24* encodes a protein involved in morphological and functional maturation of neuronal circuitry.^(^ ^67^ ^)^ This indicates that osteocytes may possess a phenotype in common with neurons. Shared features between osteocytic and neuronal connectivity have been demonstrated.^(^ ^68^ ^)^ Moreover, osteocyte transcriptome signature is enriched for genes regulating neuronal network formation, suggesting this program is important in osteocyte communication.^(^ ^69^ ^)^ *TBC1D24* mutation causes dominant hearing loss.^(^ ^70^ ^)^ These DEGs (*EGR1*, *FAF1*, *H3F3B*, *PAN2*, *RNF213*, *SAMD4A*, and *TBC1D24*) we found at 6 hours post‐culture suggest that osteocytes not only orchestrate osteoclastic bone resorption and osteoblastic bone formation, but also chondrogenesis and angiogenesis in response to mechanical loading. Whether the other DEGs (*ABCA11P*, *AC007663.3*, *AC012467.2*, *AC016737.1*, *AC018692.1*, *AC118344.2*, *AC120114.3*, *AL627230.1*, *ARHGEF2‐AS2*, *CAPN10‐AS1*, *HCG27*, *PAN3‐AS1*, *RPS27AP9*, *SS18*, *TMEM183B*, *ZNF252P‐AS1*, *AC026124.2*, *AL136141.1*, *MIR5094*, *OTX2P1*, *TTC28‐AS1*, and *TMEM183B*), which are not related to bone metabolism, are biologically relevant is currently unknown.

At 24 hours post‐culture, four genes related to bone metabolism were differentially expressed between unloaded and 2000 or 8000 μɛ loaded bone. *EGFEM1P* is related to lethal neonatal bone marrow failure syndrome with multiple congenital abnormalities including limb defects.^(^ ^71^ ^)^ *HOXD4* encodes transcription factors that regulate patterning, growth, and differentiation of skeletal elements during cartilage development.^(^ ^72^ ^)^ *FGFR3*, *WNT3A*, and *MMP8* expression is increased in *HOXD4*‐transgenic mouse chondrocytes.^(^ ^72^ ^)^ These genes encode proteins involved in chondrocyte proliferation and differentiation, cartilage transformation into bone, and extracellular matrix degradation.^(^ ^72^ ^)^ It has been shown that osteocytes may participate in the initiation of chondrogenesis/osteogenesis in periosteal cartilage and bone.^(^ ^73^ ^)^ These studies suggest that osteocytes might be involved in the regulation of skeletal development by affecting chondrocyte maturation and extracellular matrix remodeling. *SNORD91B* is associated with bone mineral density in the femoral neck.^(^ ^74^ ^)^ SNX9 plays a role in bone formation, tumorigenesis, and angiogenesis by mediating intracellular trafficking of ADAM9.^(^ ^75^ ^)^ FGF2, an important regulator of bone formation and mineralization, induces *SNX9* gene expression in MC3T3‐E1 preosteoblasts.^(^ ^76^ ^)^ These studies related to DEGs observed in our current study support the key regulatory role of osteocytes in bone remodeling. Our findings suggested that osteocytes orchestrate mechanical adaptation of bone by affecting chondrogenesis, vascular development, osteoblastogenesis, and extracellular matrix mineralization. Whether the other DEGs (*AC006299*.1, AC020892*.1*, *AL359546.1*, *FAM86C2P*, *LOC101928053*, *MSH5‐SAPCD1*, *MYLK‐AS1*, *AC000068.3*, *AC117503.4*, *AC130650.2*, *AL137783.1*, *AL138966.2*, *AP002812.1*, *ARNILA*, and *MKRN6P*), which are not related to bone metabolism, are biologically relevant is currently unknown.

This study investigated the effect of post‐culture time (0, 6, and 24 hours) on the transcriptome of osteocytes in response to mechanical loading at 2000 and 8000 μɛ. Only gene expression of *FAF1*, *PAN2*, *SAMD4A*, *and RNF213* was significantly upregulated or downregulated at 6 hours post‐culture after mechanical loading compared to 0 and 24 hours post‐culture. The mechanoresponse of osteocytes occurs within minutes to hours; ie, NO and prostaglandins are produced within few minutes after mechanical loading.^(^ ^19^ ^)^ Increased c‐fos expression in osteocytes occurs within 1 hours of mechanical stimulation, and lasts up to 4 hours.^(^ ^77^ ^)^ *MEPE* and *SOST* are increased, whereas *FGF23* is decreased in mechanically loaded rat ulnae at 6 hours post‐loading.^(^ ^38^ ^)^ *DMP1* mRNA expression increased in mouse alveolar osteocytes as early as 6 hours after mechanical loading and increased to the peak at day 4.^(^ ^31^ ^)^ The osteocyte response to mechanical loading was studied at three different post‐culture time points after mechanical loading, demonstrating that 6 hours post‐culture was the optimal time point to assess the expression of *FAF1*, *PAN2*, *SAMD4A*, and *RNF213*. This study might help to elucidate the sequence in which osteocyte mechanosensitive gene transcription takes place.

Differential gene expression of known mechanosensitive genes, eg, *SOST*, *COX‐2*, and *MEPE*, was not observed in the current study. Mechanical loading downregulates *SOST* gene expression, but upregulates *COX‐2* and *MEPE* expression in osteocytes in in vitro and in vivo animal models.^(^ ^22^, ^27^, ^33^ ^)^ Such changes in *SOST*, *COX‐2*, and *MEPE* expression have not been reported for human cortical bone explants. Our 3D‐mechanical loading model of human cortical bone containing osteocytes in their native matrix has been validated earlier.^(^ ^48^ ^)^ This model allowed for the first time the identification of mechanosensitive genes in human osteocytes in their native matrix using RNA‐seq. The amount of harvested bone from each donor was too limited to perform all studies on the effects of different loading magnitude (0, 2000, and 8000 μɛ) and different post‐culture time (0, 6, and 24 hours) on the response of osteocytes in their native matrix to mechanical loading. Moreover, donor differences could have obscured possible differences in gene expression. *RNF213* gene expression, assessed by real‐time PCR, was consistent with the results obtained by RNA‐seq. We were unable to confirm the significant mechanical loading‐induced changes in gene expression of the other DEGs found in our study by real‐time PCR. This could be due to the higher interdonor variability than the differences induced by mechanical loading, but probably, more importantly, to the fact that the average expression differences induced by mechanical loading were below twofold.^(^ ^78^ ^)^ Another limitation of this study was that mechanical loading was applied by three‐point bending, whereas fibular bone is predominantly under compression loading in the axial direction under physiological conditions.^(^ ^79^ ^)^ Osteocytes in fibulae are aligned along the principal mechanical loading direction, probably as an adaptation to the physiological mechanical loading.^(^ ^80^ ^)^ Therefore, the magnitude and/or direction of the strain perceived by osteocytes in the explants in our study might slightly differ from the in vivo situation. However, the mechanism of mechanosensing and mechanotransduction by osteocytes in our 3D‐mechanical loading model and in vivo is still similar.

In conclusion, in human bone explants containing osteocytes embedded in their native matrix, 47 new DEGs by mechanical loading were discovered. Eleven of these genes were related to bone metabolism. RNF213 might play a role in mechanical adaptation of bone by regulating angiogenesis, which is a prerequisite for successful bone formation. The functional aspects of the genes will be further explored because they might play a role in the mechanical adaptation of bone. Our study provides new insight into the mechanism of osteocyte mechanosensation and mechanotransduction, and improves our understanding of mechanical adaptation of bone. Our results suggest target genes to study the role of osteocytes in metabolic bone diseases, thereby facilitating studies of disease pathogenesis and potential gene targeted therapy.

## Author Contributions

**Chen Zhang:** Conceptualization; formal analysis; funding acquisition; investigation; methodology; visualization; writing – original draft; writing – review and editing. **Huib W. van Essen:** Methodology; writing – review and editing. **Daoud Sie:** Formal analysis; methodology; writing – review and editing. **Dimitra Micha:** Writing – review and editing. **Gerard Pals:** Writing – review and editing. **Jenneke Klein‐Nulend:** Conceptualization; funding acquisition; methodology; project administration; resources; supervision; validation; writing – review and editing. **Nathalie Bravenboer:** Conceptualization; data curation; funding acquisition; methodology; project administration; resources; supervision; validation; writing – review and editing.

## Conflict of Interest

The authors have no conflicts of interest to declare that are relevant to the content of this article.

## Ethics Approval Statement

Approval was obtained from the ethics committee of the Amsterdam University Medical Centers (2016.105). The procedures used in this study adhere to the tenets of the Declaration of Helsinki.

## Patient Consent Statement

Patient consent was obtained.

## Supporting information


**Table S1.** List of DEGs between unloaded (0 μɛ) and 2000 μɛ loaded bone or between unloaded (0 μɛ) and 8000 μɛ loaded bone at 0, 6, and 24 hours post‐culture.
**Table S2.** KEGG pathways of the DEGs between unloaded (0 μɛ) and 2000 μɛ loaded bone or between unloaded (0 μɛ) and 8000 μɛ loaded bone without post‐culture.
**Table S3.** KEGG pathways of the DEGs between unloaded (0 μɛ) and 2000 μɛ loaded bone or between unloaded (0 μɛ) and 8000 μɛ loaded bone with 6 hours post‐culture.
**Table S4.** KEGG pathways of the DEGs between unloaded (0 μɛ) and 2000 μɛ loaded bone or between unloaded (0 μɛ) and 8000 μɛ loaded bone with 24 hours post‐culture.
**Fig. S1.** Mechanical loading at 2000 or 8000 μɛ did not significantly affect the gene expression of *SOST*, *COX‐2*, and *MEPE* at 0, 6, or 24 hours post‐culture measured by RNA‐seq. Gene expression of *SOST* (*A–C*), *COX‐2* (*D–F*), and *MEPE* (*G–I*) in osteocytes in unloaded (0 μɛ), 2000 μɛ, and 8000 μɛ loaded bone with 0, 6, and 24 hours post‐culture. Each dot indicates data from one donor. n = 4.
**Fig. S2.** Mechanical loading at 2000 or 8000 μɛ did not significantly affect the gene expression of *SOST*, *COX‐2*, and *MEPE* at 0, 6, or 24 hours post‐culture measured by real‐time PCR. Gene expression of *SOST* (*A–C*), *COX‐2* (*D–F*), and *MEPE* (*G–I*) in osteocytes in unloaded (0 μɛ), 2000 μɛ, and 8000 μɛ loaded bone with 0, 6, and 24 hours post‐culture. Each dot indicates data from one donor. 0 hours, 0, 8000 μɛ, n = 6; 0 hours, 2000 μɛ, n = 7; 6 hours, 0μɛ, n = 6; 6 hours, 6 hours, 2000, 8000 μɛ, n = 8; 24 hours, 0, 2000 μɛ, n = 6; 24 hours, 8000 μɛ, n = 7.

## Data Availability

The data discussed in this publication have been deposited in NCBI's Gene Expression Omnibus^(^ ^81^ ^)^ and are accessible through GEO Series accession number GSE220630 (https://www.ncbi.nlm.nih.gov/geo/query/acc.cgi?acc=GSE220630).
