# Supplementary material for: Mapping the Response of Human Osteocytes in Native Matrix to Mechanical Loading Using RNA Sequencing
Source: JBMR Plus. 2023 Feb 21;7(4):e10721. doi: 10.1002/jbm4.10721 (PMC10097643; doi:10.1002/jbm4.10721)
Supplement: Supplementary file 1 — Table S1. List of DEGs between unloaded (0 μɛ) and 2000 μɛ loaded bone or between unloaded (0 μɛ) and 8000 μɛ loaded bone at 0, 6, and 24 hours post‐culture. Table S2. KEGG pathways of the DEGs between unloaded (0 μɛ) and 2000 μɛ loaded bone or between unloaded (0 μɛ) and 8000 μɛ loaded bone without post‐culture. Table S3. KEGG pathways of the DEGs between unloaded (0 μɛ) and 2000 μɛ loaded bone or between unloaded (0 μɛ) and 8000 μɛ loaded bone with 6 hours post‐culture. Table S4. KEGG pathways of the DEGs between unloaded (0 μɛ) and 2000 μɛ loaded bone or between unloaded (0 μɛ) and 8000 μɛ loaded bone with 24 hours post‐culture. Fig. S1. Mechanical loading at 2000 or 8000 μɛ did not significantly affect the gene expression of SOST, COX‐2, and MEPE at 0, 6, or 24 hours post‐culture measured by RNA‐seq. Gene expression of SOST (A–C), COX‐2 (D–F), and MEPE (G–I) in osteocytes in unloaded (0 μɛ), 2000 μɛ, and 8000 μɛ loaded bone with 0, 6, and 24 hours post‐culture. Each dot indicates data from one donor. n = 4. Fig. S2. Mechanical loading at 2000 or 8000 μɛ did not significantly affect the gene expression of SOST, COX‐2, and MEPE at 0, 6, or 24 hours post‐culture measured by real‐time PCR. Gene expression of SOST (A–C), COX‐2 (D–F), and MEPE (G–I) in osteocytes in unloaded (0 μɛ), 2000 μɛ, and 8000 μɛ loaded bone with 0, 6, and 24 hours post‐culture. Each dot indicates data from one donor. 0 hours, 0, 8000 μɛ, n = 6; 0 hours, 2000 μɛ, n = 7; 6 hours, 0μɛ, n = 6; 6 hours, 6 hours, 2000, 8000 μɛ, n = 8; 24 hours, 0, 2000 μɛ, n = 6; 24 hours, 8000 μɛ, n = 7. [file JBM4-7-e10721-s001.docx]

**Supplemental Table 1.** List of DEGs between unloaded (0 µɛ) and 2000 µɛ loaded bone or between unloaded (0 µɛ) and 8000 µɛ loaded bone at 0, 6, and 24 h post-culture.

| # | 0 h post-culture | | 6 h post-culture | | 24 h post-culture | |
| --- | --- | --- | --- | --- | --- | --- |
|  | DEGs between 0 µɛ vs 2000 µɛ | DEGs between 0 µɛ vs 8000 µɛ | DEGs between 0 µɛ vs 2000 µɛ | DEGs between 0 µɛ vs 8000 µɛ | DEGs between 0 µɛ vs 2000 µɛ | DEGs between 0 µɛ vs 8000 µɛ |
| 1 | *AC004053.1* | *AC000068.3* | *ABCA11P* | *AAAS* | *ABCA1* | *AC000068.3* |
| 2 | *AC005005.4* | *AC005034.6* | *AC005306.1* | *ABCA11P* | *AC000068.3* | *AC005225.2* |
| 3 | *AC005537.1* | *AC005514.1* | *AC005696.2* | *ABHD8* | *AC005537.1* | *AC006023.2* |
| 4 | *AC008443.1* | *AC007114.2* | *AC006020.1* | *AC000089.1* | *AC005906.3* | *AC006213.5* |
| 5 | *AC011773.3* | *AC007391.3* | *AC006042.2* | *AC003975.1* | *AC006299.1* | *AC006299.1* |
| 6 | *AC013486.1* | *AC007663.3* | *AC006207.1* | *AC004053.1* | *AC007222.2* | *AC007036.2* |
| 7 | *AC016168.4* | *AC007751.1* | *AC007663.3* | *AC004817.1* | *AC008505.1* | *AC008443.6* |
| 8 | *AC016590.3* | *AC007906.1* | *AC007878.1* | *AC005020.1* | *AC009065.8* | *AC009163.1* |
| 9 | *AC016737.1* | *AC008278.2* | *AC008752.3* | *AC005040.2* | *AC010203.2* | *AC010307.2* |
| 10 | *AC016995.1* | *AC008525.1* | *AC008764.6* | *AC005394.2* | *AC013734.1* | *AC010809.3* |
| 11 | *AC018692.1* | *AC008752.3* | *AC008786.1* | *AC005514.1* | *AC018628.2* | *AC010834.1* |
| 12 | *AC018865.2* | *AC009139.2* | *AC009148.1* | *AC005899.8* | *AC019197.1* | *AC011498.4* |
| 13 | *AC018926.3* | *AC009495.1* | *AC009269.4* | *AC006122.1* | *AC020612.2* | *AC012309.2* |
| 14 | *AC022960.2* | *AC009831.3* | *AC011092.3* | *AC006299.1* | *AC020892.1* | *AC012409.1* |
| 15 | *AC024361.1* | *AC011503.4* | *AC012101.2* | *AC006511.3* | *AC024405.2* | *AC015802.7* |
| 16 | *AC026124.2* | *AC012370.1* | *AC012442.1* | *AC006539.3* | *AC024560.4* | *AC016394.1* |
| 17 | *AC026954.3* | *AC013733.2* | *AC012467.2* | *AC007032.1* | *AC025062.2* | *AC016682.1* |
| 18 | *AC027544.2* | *AC015871.6* | *AC013477.1* | *AC007390.1* | *AC027601.2* | *AC019131.3* |
| 19 | *AC068279.1* | *AC022903.2* | *AC015802.3* | *AC007406.4* | *AC039056.2* | *AC020659.2* |
| 20 | *AC068473.1* | *AC034229.1* | *AC016737.1* | *AC007637.1* | *AC055717.1* | *AC020892.1* |
| 21 | *AC069148.1* | *AC037450.1* | *AC016877.1* | *AC007663.3* | *AC068413.1* | *AC022306.3* |
| 22 | *AC073840.1* | *AC040169.3* | *AC018692.1* | *AC007922.3* | *AC073349.1* | *AC022431.1* |
| 23 | *AC079193.1* | *AC060765.1* | *AC022148.2* | *AC007998.5* | *AC078923.1* | *AC026412.1* |
| 24 | *AC084125.2* | *AC062029.1* | *AC022868.1* | *AC008026.1* | *AC079341.1* | *AC026436.1* |
| 25 | *AC090984.1* | *AC068279.1* | *AC026124.2* | *AC008038.1* | *AC091978.1* | *AC026688.2* |
| 26 | *AC091179.1* | *AC073840.1* | *AC048341.2* | *AC008443.4* | *AC092118.2* | *AC027544.2* |
| 27 | *AC092473.2* | *AC087878.1* | *AC048382.2* | *AC008505.1* | *AC092634.3* | *AC068870.1* |
| 28 | *AC092652.1* | *AC090142.1* | *AC055811.3* | *AC008629.2* | *AC092896.1* | *AC074117.2* |
| 29 | *AC097059.1* | *AC091516.1* | *AC068205.2* | *AC008662.1* | *AC093838.1* | *AC078889.1* |
| 30 | *AC104117.3* | *AC093151.7* | *AC069236.1* | *AC008878.1* | *AC095040.1* | *AC079384.1* |
| 31 | *AC105398.1* | *AC096649.1* | *AC084121.2* | *AC008966.3* | *AC099335.1* | *AC087683.2* |
| 32 | *AC110285.1* | *AC098935.2* | *AC084876.2* | *AC009283.1* | *AC104333.4* | *AC090739.1* |
| 33 | *AC112253.1* | *AC104664.1* | *AC087481.1* | *AC010328.2* | *AC104394.1* | *AC091167.6* |
| 34 | *AC112504.1* | *AC108052.1* | *AC090142.1* | *AC010538.1* | *AC105129.1* | *AC096711.2* |
| 35 | *AC120036.3* | *AC108734.1* | *AC090425.3* | *AC011503.4* | *AC105450.1* | *AC103760.1* |
| 36 | *AC131009.2* | *AC108734.3* | *AC092368.3* | *AC011506.1* | *AC106782.3* | *AC117503.4* |
| 37 | *AC133552.4* | *AC110285.1* | *AC098484.2* | *AC012464.3* | *AC108019.2* | *AC129492.5* |
| 38 | *AC137590.1* | *AC116428.1* | *AC103591.2* | *AC012467.2* | *AC110285.1* | *AC130650.2* |
| 39 | *AC211486.3* | *AC130371.2* | *AC103957.2* | *AC012485.2* | *AC112236.3* | *AC244034.3* |
| 40 | *AC234775.1* | *AC133550.3* | *AC118344.2* | *AC012618.2* | *AC117383.1* | *ACTBP9* |
| 41 | *AC243732.1* | *AC136475.4* | *AC118553.2* | *AC013733.2* | *AC117503.4* | *AF131215.7* |
| 42 | *AC244034.3* | *AC140725.1* | *AC120114.3* | *AC015802.6* | *AC130650.2* | *AKAP14* |
| 43 | *ADCK3* | *AC243654.1* | *AC124248.2* | *AC016737.1* | *AC139768.1* | *AL008638.1* |
| 44 | *ADRA2B* | *AC244034.3* | *AC126175.2* | *AC018628.2* | *AC140725.1* | *AL020997.1* |
| 45 | *AL021918.5* | *ACACB* | *AC126335.1* | *AC018653.3* | *AC243654.1* | *AL031663.3* |
| 46 | *AL023775.1* | *ADRA2B* | *AC127070.3* | *AC018692.1* | *ACYP1* | *AL049776.1* |
| 47 | *AL031772.1* | *AF129075.1* | *AC127496.1* | *AC018695.6* | *ADAM33* | *AL078621.1* |
| 48 | *AL109933.5* | *AGBL3* | *AC244517.5* | *AC018809.2* | *ADAMTS20* | *AL109615.1* |
| 49 | *AL121655.1* | *AKR1D1* | *ACTR1A* | *AC020612.1* | *AF111169.4* | *AL121894.2* |
| 50 | *AL121782.1* | *AL008638.6* | *ACVR1C* | *AC020612.2* | *AL023574.1* | *AL133467.2* |
| 51 | *AL133373.2* | *AL008718.3* | *ADCY5* | *AC020633.1* | *AL033530.1* | *AL137783.1* |
| 52 | *AL139039.2* | *AL121655.1* | *ADRA1A* | *AC022306.3* | *AL034380.1* | *AL138966.2* |
| 53 | *AL158166.1* | *AL122023.1* | *AHDC1* | *AC022762.2* | *AL035413.2* | *AL158835.2* |
| 54 | *AL158823.1* | *AL136141.1* | *AK6P2* | *AC023855.1* | *AL133553.1* | *AL353704.1* |
| 55 | *AL161797.1* | *AL138716.1* | *AL023581.2* | *AC024940.2* | *AL137058.3* | *AL353705.3* |
| 56 | *AL359999.1* | *AL138889.1* | *AL031123.2* | *AC025370.2* | *AL137783.1* | *AL356019.2* |
| 57 | *AL390067.1* | *AL161797.1* | *AL035603.1* | *AC025430.1* | *AL138966.2* | *AL356218.2* |
| 58 | *AL512622.1* | *AL162724.2* | *AL049829.2* | *AC025449.1* | *AL356274.2* | *AL358072.1* |
| 59 | *AL590764.1* | *AL355612.1* | *AL096701.4* | *AC025566.1* | *AL356419.1* | *AL359546.1* |
| 60 | *ALKBH3-AS1* | *AL357054.2* | *AL117332.1* | *AC025917.1* | *AL357054.2* | *AL360182.1* |
| 61 | *AMER1* | *AL390728.2* | *AL121809.2* | *AC025946.1* | *AL358472.2* | *AL390860.1* |
| 62 | *ANAPC10P1* | *AL391650.2* | *AL122010.1* | *AC026124.2* | *AL358781.2* | *AL451085.1* |
| 63 | *AP003071.1* | *AL451085.1* | *AL133410.2* | *AC026333.4* | *AL359546.1* | *AL607028.1* |
| 64 | *C14ORF178* | *AL590764.1* | *AL136141.1* | *AC036222.2* | *AL390816.2* | *AL670729.1* |
| 65 | *C1ORF56* | *AL592464.2* | *AL139274.2* | *AC037450.1* | *AL391069.1* | *AL731702.1* |
| 66 | *C20ORF194* | *AL596442.2* | *AL161729.4* | *AC060234.3* | *AL391121.1* | *AP000477.1* |
| 67 | *C6ORF58* | *AL603914.1* | *AL353803.4* | *AC067852.4* | *AL449212.1* | *AP000577.1* |
| 68 | *CACTIN-AS1* | *AL645568.1* | *AL359091.3* | *AC068279.1* | *AL450322.2* | *AP000640.2* |
| 69 | *CLDN20* | *AP000753.2* | *AL359715.1* | *AC073264.3* | *AL691432.2* | *AP000919.2* |
| 70 | *CYP8B1* | *AP001831.1* | *AL627230.1* | *AC073342.1* | *ALG1L6P* | *AP002812.1* |
| 71 | *DLK1* | *ARMC10* | *ALG10B* | *AC073573.1* | *ANKRD30A* | *AP005717.1* |
| 72 | *DND1P1* | *ASTN2* | *ALG1L7P* | *AC073581.1* | *AP000343.1* | *AP006222.2* |
| 73 | *EEF1A1P28* | *ATP2B1* | *AMDHD2* | *AC078785.1* | *AP001462.1* | *AP006545.3* |
| 74 | *ERICH6* | *ATXN2-AS* | *AMY2A* | *AC079140.3* | *AP002748.6* | *APCDD1* |
| 75 | *FASTKD5* | *AURKBP1* | *ANKRD27* | *AC079363.1* | *AP002812.1* | *ARNILA* |
| 76 | *FOXH1* | *BEND2* | *ANKRD54* | *AC079768.1* | *AP006565.1* | *ATP5F1AP7* |
| 77 | *GCNT1P1* | *C1ORF56* | *ANP32D* | *AC079949.5* | *ARNILA* | *BX255925.2* |
| 78 | *H2AC10P* | *C20ORF24* | *ANP32E* | *AC084824.4* | *ATXN7L1* | *C2ORF66* |
| 79 | *HFE2* | *C21ORF62* | *AP000769.1* | *AC087257.1* | *BRDT* | *CHCHD10* |
| 80 | *HIST2H2AB* | *C4ORF45* | *AP001029.1* | *AC087633.2* | *C15ORF53* | *CLCC1* |
| 81 | *HMGB1P19* | *CD300LG* | *AP001284.1* | *AC090114.1* | *C20ORF27* | *CNNM2* |
| 82 | *IFT74-AS1* | *CDHR4* | *AP001831.1* | *AC090503.2* | *C7ORF65* | *CPA4* |
| 83 | *IGHV4OR15-8* | *CFAP73* | *AP003071.5* | *AC092597.1* | *CEP83-AS1* | *CPEB2-AS1* |
| 84 | *IL5* | *CFL1P3* | *AP005205.2* | *AC092745.3* | *CLDN6* | *CREG2* |
| 85 | *KBTBD13* | *CNTN6* | *ARHGEF2-AS2* | *AC092910.3* | *CNN2P12* | *CWH43* |
| 86 | *KIAA1143P1* | *CREG1* | *ARID1A* | *AC093151.7* | *CNTF* | *DDR1* |
| 87 | *KTI12* | *DLK1* | *ASCC3* | *AC096751.1* | *CNTN6* | *DNAJC3-AS1* |
| 88 | *LAMA5-AS1* | *DND1P1* | *ASS1P3* | *AC097634.1* | *COQ3* | *EFL1P1* |
| 89 | *LGI1* | *DPYD-AS1* | *ATF5* | *AC097652.1* | *DBIP1* | *EGFEM1P* |
| 90 | *LINC01210* | *ELL3* | *ATP6V0E1P2* | *AC099560.2* | *DLK1* | *FAM86C2P* |
| 91 | *LINC01871* | *FLJ42102* | *BLCAP* | *AC100871.1* | *DLX3* | *FAM87A* |
| 92 | *LMX1B* | *FN3K* | *BTC* | *AC104447.1* | *DNAH8-AS1* | *FOXB1* |
| 93 | *LOC100128531* | *GAPDHP27* | *C10ORF35* | *AC104561.4* | *DPYD-AS1* | *FOXG1-AS1* |
| 94 | *LOC100505824* | *GLOD5* | *CAMK2N2* | *AC104964.3* | *EGFEM1P* | *GEMIN6* |
| 95 | *LOC102723809* | *HMGB1P19* | *CAPN10-AS1* | *AC106795.5* | *FABP7* | *GPD1L* |
| 96 | *LOC102724612* | *HNRNPA1P45* | *CASC20* | *AC108052.1* | *FAF2* | *GSX2* |
| 97 | *LOC102724919* | *IGBP1-AS2* | *CASC9* | *AC108463.3* | *FAM86C2P* | *GTF2H3* |
| 98 | *MAPK6P6* | *IL21-AS1* | *CASKIN2* | *AC109347.1* | *FCGR3B* | *H3P24* |
| 99 | *MIR29A* | *KIAA0408* | *CCDC122* | *AC109454.1* | *FLT3* | *HES4* |
| 100 | *MIR5094* | *KLF3P1* | *CCNQP3* | *AC110749.1* | *GALNTL6* | *HMSD* |
| 101 | *MIR558* | *LGI3* | *CDC42EP3P1* | *AC110792.3* | *GRIN2A* | *HOXD4* |
| 102 | *NFATC3* | *LINC00592* | *CDK2AP2P2* | *AC110795.1* | *HIRIP3* | *HSPB3* |
| 103 | *NPBWR1* | *LINC00661* | *CEP83-AS1* | *AC112206.1* | *HIST1H2AJ* | *HYAL4* |
| 104 | *NPFFR1* | *LINC01208* | *CFAP54* | *AC112504.1* | *HIST1H4I* | *KCNIP1* |
| 105 | *OR4P4* | *LINC01285* | *CHCHD6* | *AC113382.2* | *HIST2H2BE* | *KDELC1P1* |
| 106 | *OR8D1* | *LINC01518* | *CORO7* | *AC113935.1* | *HMGN2P5* | *KLK11* |
| 107 | *OTX2P1* | *LINC01852* | *CROCC2* | *AC118344.2* | *HOXD4* | *KLLN* |
| 108 | *OXLD1* | *LINC02822* | *CSP2* | *AC120114.3* | *HSPE1P26* | *LINC00330* |
| 109 | *PACSIN1* | *LOC101927438* | *CXORF40B* | *AC121338.2* | *HYKK* | *LINC00454* |
| 110 | *PCDHGB9P* | *LOC102724153* | *DDX47* | *AC125807.1* | *IFNLR1* | *LINC02599* |
| 111 | *PGM5P4* | *LRRN2* | *DPP4-DT* | *AC126773.4* | *LINC00365* | *LINC02613* |
| 112 | *PMS2P10* | *MIR573* | *DPY19L2P4* | *AC127496.6* | *LINC00862* | *LINC02869* |
| 113 | *PMS2P9* | *MT1P3* | *DUSP3* | *AC127521.1* | *LINC01141* | *LOC100507250* |
| 114 | *POM121L14P* | *NACAP2* | *EFCAB7* | *AC133134.1* | *LINC02378* | *LOC101927934* |
| 115 | *POM121L8P* | *NAT2* | *EGR1* | *AC138150.1* | *LINC02513* | *LOC101928053* |
| 116 | *PPP1R26P1* | *OR10A2* | *EHBP1* | *AC209007.1* | *LINC02551* | *LOC101929464* |
| 117 | *PSME2P1* | *OR51B5* | *EHD1* | *AC234781.2* | *LINC02809* | *LOC102724053* |
| 118 | *PXDNL* | *OR5AM1P* | *EIF3J-DT* | *AC242842.1* | *LINC02847* | *LPA* |
| 119 | *RBM39P1* | *OR6M1* | *EIPR1-IT1* | *ACER1* | *LMOD1* | *MC5R* |
| 120 | *RN7SL505P* | *OTX2P1* | *ELMSAN1* | *ACER3* | *LOC101928053* | *MGC12916* |
| 121 | *RNU2-11P* | *PCAT14* | *EMC8* | *ACOX1* | *LOC101928514* | *MINA* |
| 122 | *RNU6-1176P* | *PMS2P9* | *ERF* | *ADAMTS20* | *LOC101928682* | *MIR3120* |
| 123 | *RORC* | *PPIAP70* | *ESRRA* | *ADCK3* | *LOC101928978* | *MIR4755* |
| 124 | *RPL17P44* | *PPP1R35* | *EXOC6B* | *ADSSL1* | *LOC101929099* | *MKRN6P* |
| 125 | *RPL21P37* | *PRR13P1* | *FAF1* | *AF129075.1* | *LOC101929745* | *MRE11P1* |
| 126 | *RPL21P39* | *RBM12B-AS1* | *FAM134C* | *AF235103.3* | *LOC105274304* | *MSH5-SAPCD1* |
| 127 | *RPL5P4* | *RCSD1* | *FAM58A* | *AGFG1* | *LOC105375483* | *MT3* |
| 128 | *RPS3AP15* | *RN7SL403P* | *FAM86GP* | *AGTRAP* | *LOC105378272* | *MYLK-AS1* |
| 129 | *RPS3AP30* | *RNF222* | *FAR2P2* | *AIP* | *LOC284865* | *MYO1H* |
| 130 | *RPS3AP31* | *RPL21P127* | *FBXL18* | *AIRE* | *LOC285626* | *NPFFR1* |
| 131 | *RPS3AP35* | *RPS5P8* | *FDPSP2* | *AKR1D1* | *LOC440910* | *NRAD1* |
| 132 | *RPS6P21* | *SAMD13* | *FDPSP7* | *AL020997.3* | *LRP2BP* | *PCDHA4* |
| 133 | *RPSAP13* | *SEC14L3* | *FERMT2* | *AL021918.5* | *LRRC16B* | *PCDHAC2* |
| 134 | *SCGB2B2* | *SLC25A30-AS1* | *FIGNL2* | *AL033543.1* | *MAP2K6* | *PGK1P2* |
| 135 | *SIRT4* | *SLC25A3P2* | *FUNDC2P2* | *AL049695.1* | *MIR122HG* | *PKD1L2* |
| 136 | *SLC25A47* | *SLC6A14* | *FZR1* | *AL078621.1* | *MIR1302-9HG* | *PPM1K* |
| 137 | *SRD5A2* | *SLC6A6P1* | *GABRA5* | *AL109615.1* | *MIR3663HG* | *RCC2P6* |
| 138 | *SSMEM1* | *SLC7A2-IT1* | *GCFC2* | *AL117692.1* | *MIR4500HG* | *RN7SL587P* |
| 139 | *SSX4B* | *SNORA49* | *GLRX5P1* | *AL122001.1* | *MKRN6P* | *RN7SL663P* |
| 140 | *TMSB15B-AS1* | *TCF15* | *GPBAR1* | *AL132640.2* | *MLANA* | *RN7SL851P* |
| 141 | *TNFRSF10D* | *THAP10* | *GPD2* | *AL132709.8* | *MLIP-IT1* | *RNU6-98P* |
| 142 | *TRGV8* | *TMEM274P* | *GRAMD1C* | *AL132857.1* | *MSH5-SAPCD1* | *RPL35P6* |
| 143 | *USP12PX* | *TTC7B* | *GUCD1* | *AL133520.1* | *MYLK-AS1* | *RPL7AP60* |
| 144 | *USP12PY* | *TUBB4AP1* | *H3F3B* | *AL136141.1* | *NCOR1P2* | *RPS15AP29* |
| 145 | *USP38* | *TVP23A* | *HCG27* | *AL136964.1* | *OLIG3* | *RPSAP36* |
| 146 | *VINAC1P* | *UBE2Q2P12* | *HINT3* | *AL137918.1* | *OPALIN* | *SMOX* |
| 147 | *VTI1A* | *WDR82P2* | *HOXA11* | *AL138895.1* | *OR10G3* | *SNORD91B* |
| 148 | *Z92544.1* | *XPC-AS1* | *HOXC11* | *AL139100.1* | *OTX2P1* | *SNX9* |
| 149 |  |  | *HUS1B* | *AL139317.3* | *PAICSP3* | *TRAPPC11* |
| 150 |  |  | *HYAL1* | *AL139424.2* | *PCNPP1* | *TRBV23-1* |
| 151 |  |  | *IL12A* | *AL158166.1* | *PDF* | *TRIM31* |
| 152 |  |  | *INO80B* | *AL354928.1* | *PIP* | *TSNAX-DISC1* |
| 153 |  |  | *KERA* | *AL355076.2* | *POLD1* | *TVP23CP1* |
| 154 |  |  | *KLF10* | *AL356108.1* | *POU6F2* | *UBALD2* |
| 155 |  |  | *KLF2* | *AL356274.2* | *PRKACG* | *VDAC1P5* |
| 156 |  |  | *KLF9* | *AL356419.1* | *PRSS8* | *VPS9D1-AS1* |
| 157 |  |  | *LEP* | *AL358072.1* | *RIMBP3* | *Z83836.1* |
| 158 |  |  | *LINC00847* | *AL359762.3* | *RIMBP3B* | *ZIC5* |
| 159 |  |  | *LINC01888* | *AL359881.1* | *RN7SL833P* | *ZMYM1* |
| 160 |  |  | *LOC100128239* | *AL359922.3* | *RN7SL861P* |  |
| 161 |  |  | *LOC100996447* | *AL360182.1* | *RNASEK* |  |
| 162 |  |  | *LOC101926980* | *AL450345.1* | *RNF222* |  |
| 163 |  |  | *LOC101929541* | *AL451074.2* | *RNU4-40P* |  |
| 164 |  |  | *LOC101929710* | *AL513210.1* | *RNU6-50P* |  |
| 165 |  |  | *LOC105373525* | *AL513365.1* | *RPL23AP6* |  |
| 166 |  |  | *LOC146880* | *AL583859.2* | *RPL23AP81* |  |
| 167 |  |  | *LOC286238* | *AL590399.2* | *RPS2P52* |  |
| 168 |  |  | *LRRFIP2* | *AL590867.2* | *RPSAP51* |  |
| 169 |  |  | *LYRM7* | *AL591030.1* | *RSF1-IT1* |  |
| 170 |  |  | *MAPK7* | *AL627230.1* | *RUSC1-AS1* |  |
| 171 |  |  | *MARCH8* | *AL645608.7* | *SEC14L3* |  |
| 172 |  |  | *MBNL3* | *AL713852.2* | *SLC6A13* |  |
| 173 |  |  | *MED28P7* | *ALDH1A1* | *SNORA74B* |  |
| 174 |  |  | *METTL10* | *ALG12* | *SNORA79B* |  |
| 175 |  |  | *METTL21A* | *ALG9* | *SNORD100* |  |
| 176 |  |  | *MIOS* | *ALPK1* | *SNORD14A* |  |
| 177 |  |  | *MIR122HG* | *ANAPC10P1* | *SNORD72* |  |
| 178 |  |  | *MIR5094* | *ANKRD42* | *SNORD91B* |  |
| 179 |  |  | *MIR6859-1* | *AP000281.1* | *SNX9* |  |
| 180 |  |  | *MOB3C* | *AP002371.1* | *SPA17P1* |  |
| 181 |  |  | *MRPS30-DT* | *AP002373.1* | *SPON1-AS1* |  |
| 182 |  |  | *MT-TF* | *AP002373.2* | *SSX1* |  |
| 183 |  |  | *MT-TT* | *AP006545.2* | *SUMO4* |  |
| 184 |  |  | *MTIF2P1* | *AP4E1* | *TCN1* |  |
| 185 |  |  | *NBEA* | *APOOL* | *TIMM17BP1* |  |
| 186 |  |  | *NBR1* | *APOOP1* | *TMEM246-AS1* |  |
| 187 |  |  | *NTN3* | *ARAP1* | *TMTC4* |  |
| 188 |  |  | *NUTM2HP* | *ARHGEF2-AS2* | *TRIM68* |  |
| 189 |  |  | *OR51B5* | *ASGR1* | *ZNF209P* |  |
| 190 |  |  | *OTUD6B-AS1* | *ATAD2B* |  |  |
| 191 |  |  | *OTX2P1* | *ATF6B* |  |  |
| 192 |  |  | *OXNAD1* | *ATG7* |  |  |
| 193 |  |  | *PAK4* | *ATHL1* |  |  |
| 194 |  |  | *PAN2* | *ATP2B1* |  |  |
| 195 |  |  | *PAN3-AS1* | *ATP5MFP5* |  |  |
| 196 |  |  | *PCCA* | *ATP5SL* |  |  |
| 197 |  |  | *PDE8A* | *ATP6V0A1* |  |  |
| 198 |  |  | *PGGT1BP1* | *ATP8A2P3* |  |  |
| 199 |  |  | *PIM1* | *ATXN7L2* |  |  |
| 200 |  |  | *PPP1R26-AS1* | *AVL9* |  |  |
| 201 |  |  | *PTPN2P2* | *B3GALNT2* |  |  |
| 202 |  |  | *PUS10* | *BCAS3* |  |  |
| 203 |  |  | *RAB17* | *BCAT2* |  |  |
| 204 |  |  | *RAB43* | *BCOR* |  |  |
| 205 |  |  | *RAB43P1* | *BEND2* |  |  |
| 206 |  |  | *RAD50* | *BLVRB* |  |  |
| 207 |  |  | *RDXP1* | *BMP2K* |  |  |
| 208 |  |  | *RHBDL3* | *BMPER* |  |  |
| 209 |  |  | *RHOQP2* | *BNIP1* |  |  |
| 210 |  |  | *RN7SL434P* | *BRE* |  |  |
| 211 |  |  | *RNASEL* | *BTF3P10* |  |  |
| 212 |  |  | *RNF213* | *BUD13P1* |  |  |
| 213 |  |  | *RNU6-759P* | *BX571846.1* |  |  |
| 214 |  |  | *RNY1P9* | *C12ORF42* |  |  |
| 215 |  |  | *RPL12P27* | *C12ORF57* |  |  |
| 216 |  |  | *RPL17P41* | *C14ORF159* |  |  |
| 217 |  |  | *RPL35P4* | *C16ORF52* |  |  |
| 218 |  |  | *RPL6P9* | *C16ORF62* |  |  |
| 219 |  |  | *RPL9P28* | *C17ORF49* |  |  |
| 220 |  |  | *RPS27AP9* | *C18ORF32* |  |  |
| 221 |  |  | *RPS6P21* | *C20ORF194* |  |  |
| 222 |  |  | *SAMD4A* | *C6* |  |  |
| 223 |  |  | *SH3BP5L* | *C6ORF58* |  |  |
| 224 |  |  | *SIRPAP1* | *CAB39* |  |  |
| 225 |  |  | *SLC9A1* | *CABIN1* |  |  |
| 226 |  |  | *SMC2-AS1* | *CACFD1* |  |  |
| 227 |  |  | *SNAP23* | *CACUL1* |  |  |
| 228 |  |  | *SNORA33* | *CALCOCO1* |  |  |
| 229 |  |  | *SNORD100* | *CALML3* |  |  |
| 230 |  |  | *SOCS5P3* | *CAPN10-AS1* |  |  |
| 231 |  |  | *SPECC1L* | *CAPZA1* |  |  |
| 232 |  |  | *SS18* | *CC2D1A* |  |  |
| 233 |  |  | *ST18* | *CCDC129* |  |  |
| 234 |  |  | *ST7-AS2* | *CCDC61* |  |  |
| 235 |  |  | *ST8SIA2* | *CDH16* |  |  |
| 236 |  |  | *STARD13-IT1* | *CDHR4* |  |  |
| 237 |  |  | *STON2* | *CDK8* |  |  |
| 238 |  |  | *SUB1P3* | *CEP57L1* |  |  |
| 239 |  |  | *SUGT1* | *CFAP298-TCP10L* |  |  |
| 240 |  |  | *SUGT1P2* | *CILP* |  |  |
| 241 |  |  | *TATDN2* | *CMTR2* |  |  |
| 242 |  |  | *TBC1D24* | *CNEP1R1* |  |  |
| 243 |  |  | *TCP10* | *CNOT6* |  |  |
| 244 |  |  | *THADA* | *COMMD2* |  |  |
| 245 |  |  | *TJP2* | *COPZ1* |  |  |
| 246 |  |  | *TMEM183B* | *COX7A2* |  |  |
| 247 |  |  | *TMEM86A* | *CROCCP3* |  |  |
| 248 |  |  | *TMTC2* | *CRY1* |  |  |
| 249 |  |  | *TOR4A* | *CSRNP1* |  |  |
| 250 |  |  | *TRIM47* | *CTIF* |  |  |
| 251 |  |  | *TRIM60P17* | *CYP2F1* |  |  |
| 252 |  |  | *TRPV3* | *DACH2* |  |  |
| 253 |  |  | *TTBK2* | *DAND5* |  |  |
| 254 |  |  | *TTC28-AS1* | *DCAF8L2* |  |  |
| 255 |  |  | *TUBB4AP1* | *DCTN1* |  |  |
| 256 |  |  | *UBE2E2-AS1* | *DCUN1D4* |  |  |
| 257 |  |  | *UBE2I* | *DCUN1D5* |  |  |
| 258 |  |  | *UBE2L3* | *DEAF1* |  |  |
| 259 |  |  | *UBE2Q2P12* | *DENND1A* |  |  |
| 260 |  |  | *UCK1* | *DENND4C* |  |  |
| 261 |  |  | *UMODL1-AS1* | *DIRC2* |  |  |
| 262 |  |  | *UNQ6494* | *DLK1* |  |  |
| 263 |  |  | *VASN* | *DNAAF2* |  |  |
| 264 |  |  | *VPS13B* | *DNAL1* |  |  |
| 265 |  |  | *WDFY3-AS2* | *DNAL4* |  |  |
| 266 |  |  | *WIPI2* | *DOPEY1* |  |  |
| 267 |  |  | *WWTR1* | *E2F5* |  |  |
| 268 |  |  | *Z82243.1* | *EEF1A1* |  |  |
| 269 |  |  | *Z95331.1* | *EEF1A1P10* |  |  |
| 270 |  |  | *Z98257.1* | *EEF1A1P11* |  |  |
| 271 |  |  | *ZMAT1* | *EEF1A1P12* |  |  |
| 272 |  |  | *ZNF252P-AS1* | *EEF1A1P13* |  |  |
| 273 |  |  | *ZNF433* | *EEF1A1P16* |  |  |
| 274 |  |  | *ZNF454-DT* | *EEF1A1P19* |  |  |
| 275 |  |  | *ZNF623* | *EEF1A1P22* |  |  |
| 276 |  |  | *ZNF733P* | *EEF1A1P25* |  |  |
| 277 |  |  | *ZNF781* | *EEF1A1P38* |  |  |
| 278 |  |  | *ZSCAN2* | *EEF1A1P4* |  |  |
| 279 |  |  |  | *EEF1A1P5* |  |  |
| 280 |  |  |  | *EEF1A1P6* |  |  |
| 281 |  |  |  | *EEF1A1P8* |  |  |
| 282 |  |  |  | *EEF1A1P9* |  |  |
| 283 |  |  |  | *EFNA3* |  |  |
| 284 |  |  |  | *EGR1* |  |  |
| 285 |  |  |  | *EIF3L* |  |  |
| 286 |  |  |  | *EIF3LP3* |  |  |
| 287 |  |  |  | *EIF4BP3* |  |  |
| 288 |  |  |  | *EIF4BP9* |  |  |
| 289 |  |  |  | *EML4* |  |  |
| 290 |  |  |  | *ENDOG* |  |  |
| 291 |  |  |  | *EPHA7* |  |  |
| 292 |  |  |  | *EPOR* |  |  |
| 293 |  |  |  | *ERCC4* |  |  |
| 294 |  |  |  | *ERVK13-1* |  |  |
| 295 |  |  |  | *ETV2* |  |  |
| 296 |  |  |  | *EXOC6* |  |  |
| 297 |  |  |  | *FAF1* |  |  |
| 298 |  |  |  | *FAM214A* |  |  |
| 299 |  |  |  | *FAM230G* |  |  |
| 300 |  |  |  | *FAM35A* |  |  |
| 301 |  |  |  | *FANCF* |  |  |
| 302 |  |  |  | *FANK1* |  |  |
| 303 |  |  |  | *FARS2* |  |  |
| 304 |  |  |  | *FBXO18* |  |  |
| 305 |  |  |  | *FBXO22* |  |  |
| 306 |  |  |  | *FBXW2* |  |  |
| 307 |  |  |  | *FLVCR1* |  |  |
| 308 |  |  |  | *FOS* |  |  |
| 309 |  |  |  | *FOXO4* |  |  |
| 310 |  |  |  | *FSD1L* |  |  |
| 311 |  |  |  | *FUNDC2P1* |  |  |
| 312 |  |  |  | *GABRG1* |  |  |
| 313 |  |  |  | *GCKR* |  |  |
| 314 |  |  |  | *GJC3* |  |  |
| 315 |  |  |  | *GLIS1* |  |  |
| 316 |  |  |  | *GLRX2* |  |  |
| 317 |  |  |  | *GLS* |  |  |
| 318 |  |  |  | *GNPNAT1* |  |  |
| 319 |  |  |  | *GPAT4* |  |  |
| 320 |  |  |  | *GPR171* |  |  |
| 321 |  |  |  | *GPR174* |  |  |
| 322 |  |  |  | *GPR26* |  |  |
| 323 |  |  |  | *GYG2-AS1* |  |  |
| 324 |  |  |  | *H3F3B* |  |  |
| 325 |  |  |  | *H3F3C* |  |  |
| 326 |  |  |  | *HABP4* |  |  |
| 327 |  |  |  | *HAND2-AS1* |  |  |
| 328 |  |  |  | *HCG25* |  |  |
| 329 |  |  |  | *HCG27* |  |  |
| 330 |  |  |  | *HDAC6* |  |  |
| 331 |  |  |  | *HECW2* |  |  |
| 332 |  |  |  | *HMGB1P24* |  |  |
| 333 |  |  |  | *HN1* |  |  |
| 334 |  |  |  | *HNRNPKP1* |  |  |
| 335 |  |  |  | *HOMER1* |  |  |
| 336 |  |  |  | *IDH3B* |  |  |
| 337 |  |  |  | *IFT172* |  |  |
| 338 |  |  |  | *IL12A-AS1* |  |  |
| 339 |  |  |  | *INA* |  |  |
| 340 |  |  |  | *INHBA* |  |  |
| 341 |  |  |  | *JPH1* |  |  |
| 342 |  |  |  | *JRK* |  |  |
| 343 |  |  |  | *JUNB* |  |  |
| 344 |  |  |  | *KANK3* |  |  |
| 345 |  |  |  | *KATNBL1* |  |  |
| 346 |  |  |  | *KCNC4* |  |  |
| 347 |  |  |  | *KIAA0232* |  |  |
| 348 |  |  |  | *KIAA1468* |  |  |
| 349 |  |  |  | *KIF2A* |  |  |
| 350 |  |  |  | *KLC4* |  |  |
| 351 |  |  |  | *KLHL8* |  |  |
| 352 |  |  |  | *KLRA1P* |  |  |
| 353 |  |  |  | *LAMA5-AS1* |  |  |
| 354 |  |  |  | *LHX5* |  |  |
| 355 |  |  |  | *LIG4* |  |  |
| 356 |  |  |  | *LINC00276* |  |  |
| 357 |  |  |  | *LINC00392* |  |  |
| 358 |  |  |  | *LINC00639* |  |  |
| 359 |  |  |  | *LINC00682* |  |  |
| 360 |  |  |  | *LINC00861* |  |  |
| 361 |  |  |  | *LINC00879* |  |  |
| 362 |  |  |  | *LINC01150* |  |  |
| 363 |  |  |  | *LINC01208* |  |  |
| 364 |  |  |  | *LINC01297* |  |  |
| 365 |  |  |  | *LINC01359* |  |  |
| 366 |  |  |  | *LINC01375* |  |  |
| 367 |  |  |  | *LINC01795* |  |  |
| 368 |  |  |  | *LINC01905* |  |  |
| 369 |  |  |  | *LINC02354* |  |  |
| 370 |  |  |  | *LINC02741* |  |  |
| 371 |  |  |  | *LLGL2* |  |  |
| 372 |  |  |  | *LMNTD2* |  |  |
| 373 |  |  |  | *LOC100129940* |  |  |
| 374 |  |  |  | *LOC100505824* |  |  |
| 375 |  |  |  | *LOC100505853* |  |  |
| 376 |  |  |  | *LOC101927132* |  |  |
| 377 |  |  |  | *LOC101927178* |  |  |
| 378 |  |  |  | *LOC101927502* |  |  |
| 379 |  |  |  | *LOC101928978* |  |  |
| 380 |  |  |  | *LOC101929227* |  |  |
| 381 |  |  |  | *LOC101929420* |  |  |
| 382 |  |  |  | *LOC105369891* |  |  |
| 383 |  |  |  | *LOC105378405* |  |  |
| 384 |  |  |  | *LOC339529* |  |  |
| 385 |  |  |  | *LOC339874* |  |  |
| 386 |  |  |  | *LOC374443* |  |  |
| 387 |  |  |  | *LOC729867* |  |  |
| 388 |  |  |  | *LOC730100* |  |  |
| 389 |  |  |  | *LOC81691* |  |  |
| 390 |  |  |  | *LONRF1* |  |  |
| 391 |  |  |  | *LRP5* |  |  |
| 392 |  |  |  | *LSP1P3* |  |  |
| 393 |  |  |  | *LYRM2* |  |  |
| 394 |  |  |  | *MAGEA4-AS1* |  |  |
| 395 |  |  |  | *MEMO1P1* |  |  |
| 396 |  |  |  | *MESTIT1* |  |  |
| 397 |  |  |  | *MFN1* |  |  |
| 398 |  |  |  | *MFSD14B* |  |  |
| 399 |  |  |  | *MFSD14C* |  |  |
| 400 |  |  |  | *MFSD8* |  |  |
| 401 |  |  |  | *MIF4GD* |  |  |
| 402 |  |  |  | *MIR365A* |  |  |
| 403 |  |  |  | *MIR3976HG* |  |  |
| 404 |  |  |  | *MIR5094* |  |  |
| 405 |  |  |  | *MIR6753* |  |  |
| 406 |  |  |  | *MIS18A-AS1* |  |  |
| 407 |  |  |  | *MLLT4-AS1* |  |  |
| 408 |  |  |  | *MMP20* |  |  |
| 409 |  |  |  | *MMP7* |  |  |
| 410 |  |  |  | *MPI* |  |  |
| 411 |  |  |  | *MRGPRF-AS1* |  |  |
| 412 |  |  |  | *MRPL30* |  |  |
| 413 |  |  |  | *MRPL9* |  |  |
| 414 |  |  |  | *MRPS10* |  |  |
| 415 |  |  |  | *MRPS31P4* |  |  |
| 416 |  |  |  | *MRVI1-AS1* |  |  |
| 417 |  |  |  | *MS4A7* |  |  |
| 418 |  |  |  | *MTND5P21* |  |  |
| 419 |  |  |  | *MTSS1* |  |  |
| 420 |  |  |  | *MYH7B* |  |  |
| 421 |  |  |  | *MYL12BP1* |  |  |
| 422 |  |  |  | *MYL7* |  |  |
| 423 |  |  |  | *NAA50* |  |  |
| 424 |  |  |  | *NADSYN1* |  |  |
| 425 |  |  |  | *NBEAP5* |  |  |
| 426 |  |  |  | *NCAM2* |  |  |
| 427 |  |  |  | *NCAPD2* |  |  |
| 428 |  |  |  | *NCKIPSD* |  |  |
| 429 |  |  |  | *NDRG1* |  |  |
| 430 |  |  |  | *NDUFA13* |  |  |
| 431 |  |  |  | *NDUFS2* |  |  |
| 432 |  |  |  | *NEDD4* |  |  |
| 433 |  |  |  | *NEK9* |  |  |
| 434 |  |  |  | *NLK* |  |  |
| 435 |  |  |  | *NPHP3* |  |  |
| 436 |  |  |  | *NPM1P26* |  |  |
| 437 |  |  |  | *NPRL2* |  |  |
| 438 |  |  |  | *NR4A1* |  |  |
| 439 |  |  |  | *NUTF2P2* |  |  |
| 440 |  |  |  | *OR10J1* |  |  |
| 441 |  |  |  | *OR52A4P* |  |  |
| 442 |  |  |  | *OR52K3P* |  |  |
| 443 |  |  |  | *OR7E109P* |  |  |
| 444 |  |  |  | *OR7E46P* |  |  |
| 445 |  |  |  | *OTP* |  |  |
| 446 |  |  |  | *OTX2P1* |  |  |
| 447 |  |  |  | *PABPN1P1* |  |  |
| 448 |  |  |  | *PACSIN1* |  |  |
| 449 |  |  |  | *PAFAH1B1* |  |  |
| 450 |  |  |  | *PAN2* |  |  |
| 451 |  |  |  | *PAN3-AS1* |  |  |
| 452 |  |  |  | *PARD6B* |  |  |
| 453 |  |  |  | *PBX3* |  |  |
| 454 |  |  |  | *PFKL* |  |  |
| 455 |  |  |  | *PGAP2* |  |  |
| 456 |  |  |  | *PGM1* |  |  |
| 457 |  |  |  | *PHC1P1* |  |  |
| 458 |  |  |  | *PICK1* |  |  |
| 459 |  |  |  | *PIP* |  |  |
| 460 |  |  |  | *PIP5KL1* |  |  |
| 461 |  |  |  | *PLA2G6* |  |  |
| 462 |  |  |  | *PLAUR* |  |  |
| 463 |  |  |  | *POMGNT2* |  |  |
| 464 |  |  |  | *POU3F3* |  |  |
| 465 |  |  |  | *PPARGC1B* |  |  |
| 466 |  |  |  | *PPIE* |  |  |
| 467 |  |  |  | *PPP1R26P1* |  |  |
| 468 |  |  |  | *PPP1R9A* |  |  |
| 469 |  |  |  | *PPP3R1* |  |  |
| 470 |  |  |  | *PREX1* |  |  |
| 471 |  |  |  | *PRR27* |  |  |
| 472 |  |  |  | *PRUNE* |  |  |
| 473 |  |  |  | *PSME4* |  |  |
| 474 |  |  |  | *QRFP* |  |  |
| 475 |  |  |  | *RAB10* |  |  |
| 476 |  |  |  | *RAB12* |  |  |
| 477 |  |  |  | *RAB30-AS1* |  |  |
| 478 |  |  |  | *RABEP1* |  |  |
| 479 |  |  |  | *RAC3* |  |  |
| 480 |  |  |  | *RAD1* |  |  |
| 481 |  |  |  | *RALA* |  |  |
| 482 |  |  |  | *RALGPS2* |  |  |
| 483 |  |  |  | *RAP1B* |  |  |
| 484 |  |  |  | *RAP2A* |  |  |
| 485 |  |  |  | *RASA2* |  |  |
| 486 |  |  |  | *RASSF1-AS1* |  |  |
| 487 |  |  |  | *RBBP4P1* |  |  |
| 488 |  |  |  | *REV1* |  |  |
| 489 |  |  |  | *RFPL4A* |  |  |
| 490 |  |  |  | *RGL3* |  |  |
| 491 |  |  |  | *RIMS1* |  |  |
| 492 |  |  |  | *RN7SKP23* |  |  |
| 493 |  |  |  | *RNF213* |  |  |
| 494 |  |  |  | *RNF26* |  |  |
| 495 |  |  |  | *RNU6-757P* |  |  |
| 496 |  |  |  | *RNU6-834P* |  |  |
| 497 |  |  |  | *ROBO3* |  |  |
| 498 |  |  |  | *RORC* |  |  |
| 499 |  |  |  | *RPL10A* |  |  |
| 500 |  |  |  | *RPL11P3* |  |  |
| 501 |  |  |  | *RPL12* |  |  |
| 502 |  |  |  | *RPL12P1* |  |  |
| 503 |  |  |  | *RPL12P13* |  |  |
| 504 |  |  |  | *RPL12P2* |  |  |
| 505 |  |  |  | *RPL12P35* |  |  |
| 506 |  |  |  | *RPL12P38* |  |  |
| 507 |  |  |  | *RPL12P8* |  |  |
| 508 |  |  |  | *RPL13A* |  |  |
| 509 |  |  |  | *RPL13AP5* |  |  |
| 510 |  |  |  | *RPL15P20* |  |  |
| 511 |  |  |  | *RPL15P3* |  |  |
| 512 |  |  |  | *RPL17P36* |  |  |
| 513 |  |  |  | *RPL18* |  |  |
| 514 |  |  |  | *RPL23* |  |  |
| 515 |  |  |  | *RPL23AP42* |  |  |
| 516 |  |  |  | *RPL23P8* |  |  |
| 517 |  |  |  | *RPL3* |  |  |
| 518 |  |  |  | *RPL34P33* |  |  |
| 519 |  |  |  | *RPL36AP16* |  |  |
| 520 |  |  |  | *RPL37* |  |  |
| 521 |  |  |  | *RPL3P4* |  |  |
| 522 |  |  |  | *RPL41* |  |  |
| 523 |  |  |  | *RPL6* |  |  |
| 524 |  |  |  | *RPL7A* |  |  |
| 525 |  |  |  | *RPL7P56* |  |  |
| 526 |  |  |  | *RPS10P2* |  |  |
| 527 |  |  |  | *RPS11* |  |  |
| 528 |  |  |  | *RPS12P5* |  |  |
| 529 |  |  |  | *RPS20* |  |  |
| 530 |  |  |  | *RPS23* |  |  |
| 531 |  |  |  | *RPS23P8* |  |  |
| 532 |  |  |  | *RPS27* |  |  |
| 533 |  |  |  | *RPS27AP9* |  |  |
| 534 |  |  |  | *RPS3* |  |  |
| 535 |  |  |  | *RPS6* |  |  |
| 536 |  |  |  | *RPS6KA3* |  |  |
| 537 |  |  |  | *RPS6P25* |  |  |
| 538 |  |  |  | *RRAS2* |  |  |
| 539 |  |  |  | *RRNAD1* |  |  |
| 540 |  |  |  | *RSBN1L* |  |  |
| 541 |  |  |  | *SAMD13* |  |  |
| 542 |  |  |  | *SAMD4A* |  |  |
| 543 |  |  |  | *SATB2* |  |  |
| 544 |  |  |  | *SDF2* |  |  |
| 545 |  |  |  | *SELENOKP2* |  |  |
| 546 |  |  |  | *SENP6* |  |  |
| 547 |  |  |  | *SETP4* |  |  |
| 548 |  |  |  | *SH3D21* |  |  |
| 549 |  |  |  | *SHLD2P3* |  |  |
| 550 |  |  |  | *SKIV2L* |  |  |
| 551 |  |  |  | *SLC30A7* |  |  |
| 552 |  |  |  | *SLC39A5* |  |  |
| 553 |  |  |  | *SLC41A2* |  |  |
| 554 |  |  |  | *SLC41A3* |  |  |
| 555 |  |  |  | *SLC45A1* |  |  |
| 556 |  |  |  | *SLC6A6P1* |  |  |
| 557 |  |  |  | *SLITRK2* |  |  |
| 558 |  |  |  | *SMARCAL1* |  |  |
| 559 |  |  |  | *SMIM15P2* |  |  |
| 560 |  |  |  | *SND1* |  |  |
| 561 |  |  |  | *SNRPD2P1* |  |  |
| 562 |  |  |  | *SNX27* |  |  |
| 563 |  |  |  | *SP110* |  |  |
| 564 |  |  |  | *SPACA3* |  |  |
| 565 |  |  |  | *SPAST* |  |  |
| 566 |  |  |  | *SRPK2* |  |  |
| 567 |  |  |  | *SS18* |  |  |
| 568 |  |  |  | *STAT6* |  |  |
| 569 |  |  |  | *STK36* |  |  |
| 570 |  |  |  | *SUOX* |  |  |
| 571 |  |  |  | *TAF13* |  |  |
| 572 |  |  |  | *TAF4* |  |  |
| 573 |  |  |  | *TBC1D22A* |  |  |
| 574 |  |  |  | *TBC1D24* |  |  |
| 575 |  |  |  | *TBCD* |  |  |
| 576 |  |  |  | *TIAL1* |  |  |
| 577 |  |  |  | *TMCO2* |  |  |
| 578 |  |  |  | *TMCO4* |  |  |
| 579 |  |  |  | *TMEM105* |  |  |
| 580 |  |  |  | *TMEM183B* |  |  |
| 581 |  |  |  | *TMEM271* |  |  |
| 582 |  |  |  | *TMEM274P* |  |  |
| 583 |  |  |  | *TMPRSS11F* |  |  |
| 584 |  |  |  | *TNFRSF21* |  |  |
| 585 |  |  |  | *TP53TG3HP* |  |  |
| 586 |  |  |  | *TPTE2P5* |  |  |
| 587 |  |  |  | *TRAF3IP1* |  |  |
| 588 |  |  |  | *TRAF4* |  |  |
| 589 |  |  |  | *TRAM2* |  |  |
| 590 |  |  |  | *TRBV23-1* |  |  |
| 591 |  |  |  | *TRIM49C* |  |  |
| 592 |  |  |  | *TSEN15* |  |  |
| 593 |  |  |  | *TSPAN31* |  |  |
| 594 |  |  |  | *TSSC1* |  |  |
| 595 |  |  |  | *TTC28-AS1* |  |  |
| 596 |  |  |  | *UBALD2* |  |  |
| 597 |  |  |  | *UBE2E2* |  |  |
| 598 |  |  |  | *UBE2G1* |  |  |
| 599 |  |  |  | *UBE2K* |  |  |
| 600 |  |  |  | *UBR3* |  |  |
| 601 |  |  |  | *UXT* |  |  |
| 602 |  |  |  | *VHL* |  |  |
| 603 |  |  |  | *VKORC1L1* |  |  |
| 604 |  |  |  | *VPS16* |  |  |
| 605 |  |  |  | *VPS52* |  |  |
| 606 |  |  |  | *WBP2* |  |  |
| 607 |  |  |  | *WDR26* |  |  |
| 608 |  |  |  | *WDR44* |  |  |
| 609 |  |  |  | *WDR70* |  |  |
| 610 |  |  |  | *WDTC1* |  |  |
| 611 |  |  |  | *XIAP-AS1* |  |  |
| 612 |  |  |  | *XPNPEP1* |  |  |
| 613 |  |  |  | *YIPF3* |  |  |
| 614 |  |  |  | *Z97832.2* |  |  |
| 615 |  |  |  | *Z97986.1* |  |  |
| 616 |  |  |  | *ZBTB37* |  |  |
| 617 |  |  |  | *ZBTB7A* |  |  |
| 618 |  |  |  | *ZEB1-AS1* |  |  |
| 619 |  |  |  | *ZNF252P-AS1* |  |  |
| 620 |  |  |  | *ZNF271P* |  |  |
| 621 |  |  |  | *ZNF284* |  |  |
| 622 |  |  |  | *ZNF302* |  |  |
| 623 |  |  |  | *ZNF429* |  |  |
| 624 |  |  |  | *ZNF436-AS1* |  |  |
| 625 |  |  |  | *ZNF529-AS1* |  |  |
| 626 |  |  |  | *ZNF546* |  |  |
| 627 |  |  |  | *ZNF572* |  |  |
| 628 |  |  |  | *ZNF658B* |  |  |
| 629 |  |  |  | *ZNF687* |  |  |
| 630 |  |  |  | *ZNF879* |  |  |
| 631 |  |  |  | *ZNF891* |  |  |

**Supplemental Table 2.** KEGG pathways of the DEGs between unloaded (0 µɛ) and 2000 µɛ loaded bone or between unloaded (0 µɛ) and 8000 µɛ loaded bone without post-culture.

| ID | Name | p-Value | Count in gene set |
| --- | --- | --- | --- |
| hsa04740 | Olfactory transduction | 0.037 | 5 of 150 |
| hsa00120 | Primary bile acid biosynthesis | 0.049 | 2 of 150 |
| hsa04022 | cGMP-PKG signaling pathway | 0.085 | 3 of 150 |

KEGG, Kyoto Encyclopedia of Genes and Genomes; ID, identifiers of KEGG pathways.

**Supplemental Table 3.** KEGG pathways of the DEGs between unloaded (0 µɛ) and 2000 µɛ loaded bone or between unloaded (0 µɛ) and 8000 µɛ loaded bone with 6 h post-culture.

| ID | Name | p-Value | Count in gene set |
| --- | --- | --- | --- |
| hsa03010 | Ribosome | 0.000 | 19 of 604 |
| hsa05171 | Coronavirus disease - COVID-19 | 0.000 | 19 of 604 |
| hsa04120 | Ubiquitin mediated proteolysis | 0.022 | 9 of 604 |
| hsa04970 | Salivary secretion | 0.072 | 6 of 604 |
| hsa05031 | Amphetamine addiction | 0.086 | 5 of 604 |

KEGG, Kyoto Encyclopedia of Genes and Genomes; ID, identifiers of KEGG pathways.

**Supplemental Table 4.** KEGG pathways of the DEGs between unloaded (0 µɛ) and 2000 µɛ loaded bone or between unloaded (0 µɛ) and 8000 µɛ loaded bone with 24 h post-culture.

| ID | Name | p-Value | Count in gene set |
| --- | --- | --- | --- |
| hsa05322 | Systemic lupus erythematosus | 0.002 | 5 of 180 |
| hsa05034 | Alcoholism | 0.006 | 5 of 180 |
| hsa04613 | Neutrophil extracellular trap formation | 0.040 | 4 of 180 |
| hsa05203 | Viral carcinogenesis | 0.048 | 4 of 180 |

KEGG, Kyoto Encyclopedia of Genes and Genomes; ID, identifiers of KEGG pathways.

**
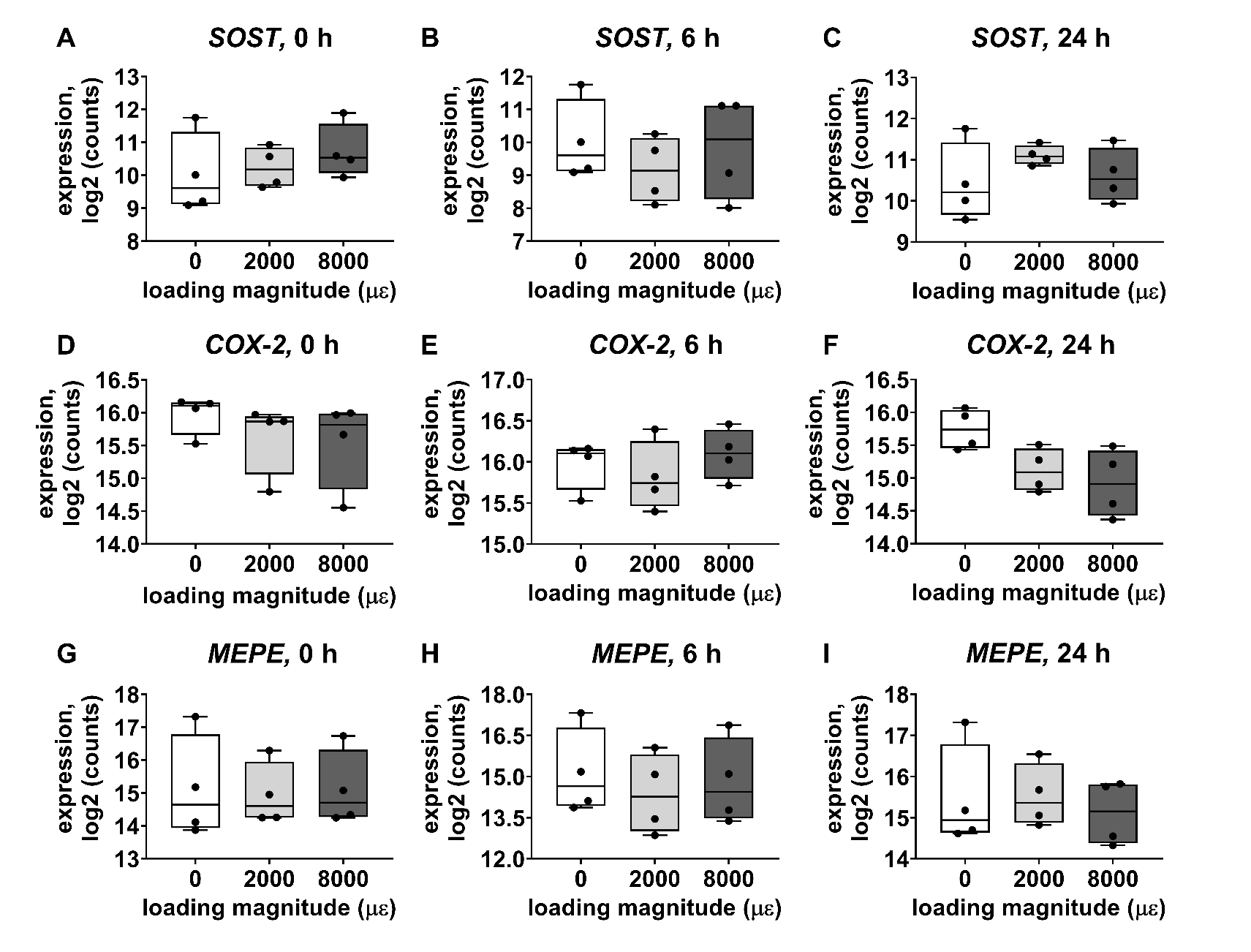
Supplemental Fig. 1** Mechanical loading at 2000 or 8000 µɛ did not significantly affect the gene expression of *SOST*, *COX-2*, and *MEPE* at 0, 6, or 24 h post-culture measured by RNA-seq. Gene expression of *SOST* (**A-C**), *COX-2* (**D-F**), and *MEPE* (**G-I**) in osteocytes in unloaded (0 µɛ), 2000 µɛ, and 8000 µɛ loaded bone with 0, 6, and 24 h post-culture. Each dot indicates data from one donor. n=4.

**
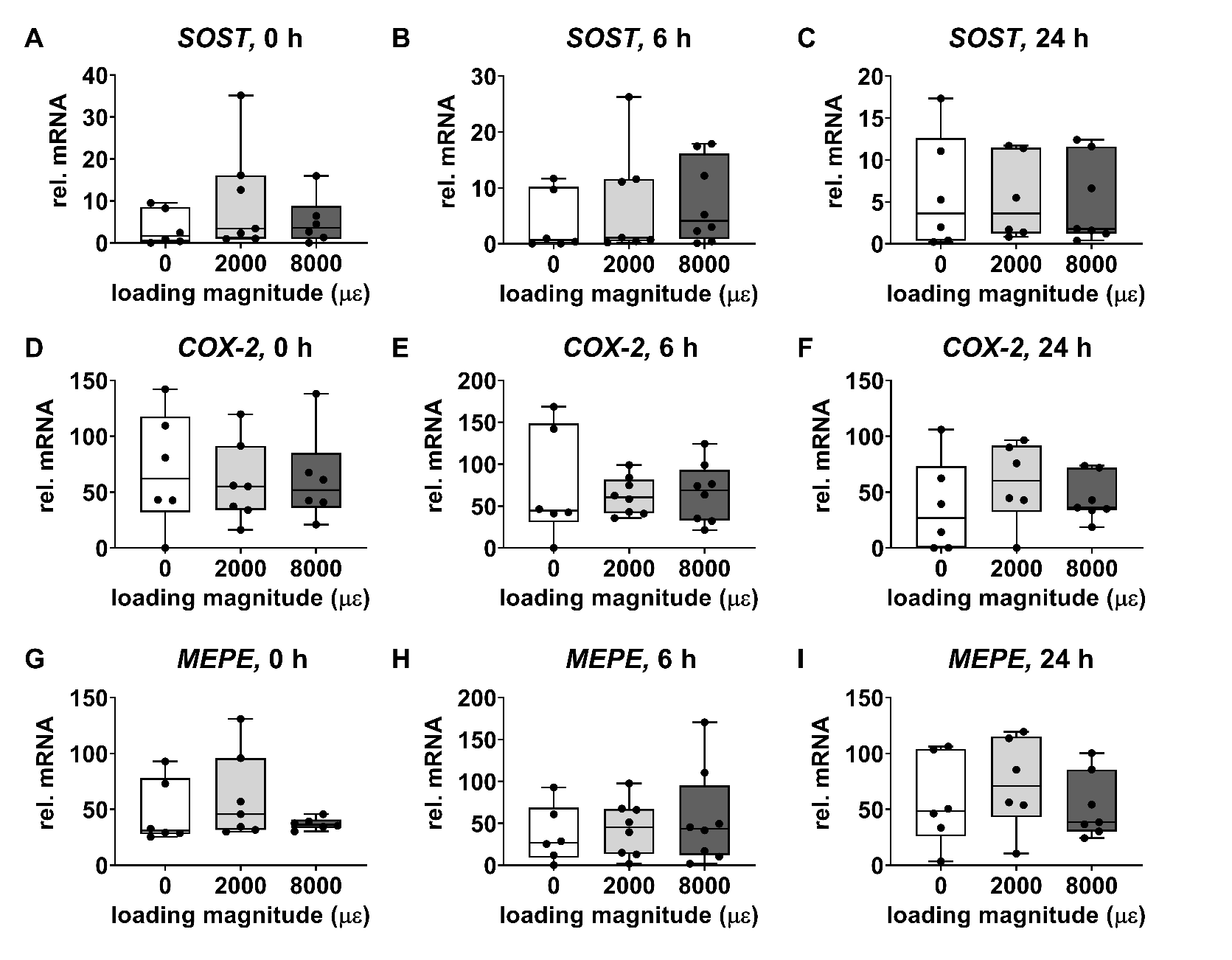
Supplemental Fig. 2** Mechanical loading at 2000 or 8000 µɛ did not significantly affect the gene expression of *SOST*, *COX-2*, and *MEPE* at 0, 6, or 24 h post-culture measured by real-time PCR. Gene expression of *SOST* (**A-C**), *COX-2* (**D-F**), and *MEPE* (**G-I**) in osteocytes in unloaded (0 µɛ), 2000 µɛ, and 8000 µɛ loaded bone with 0, 6, and 24 h post-culture. Each dot indicates data from one donor. 0 h, 0, 8000 µɛ, n=6; 0 h, 2000 µɛ, n=7; 6 h, 0µɛ, n=6; 6 h, 6 h, 2000, 8000 µɛ, n=8; 24 h, 0, 2000 µɛ, n=6; 24 h, 8000 µɛ, n=7.
